# Supplementary material for: Identifying gene targets for brain-related traits using transcriptomic and methylomic data from blood
Source: Nat Commun. 2018 Jun 11;9:2282. doi: 10.1038/s41467-018-04558-1 (PMC5995828; doi:10.1038/s41467-018-04558-1)
Supplement: Supplementary file 1 — Supplementary Information [file 41467_2018_4558_MOESM1_ESM.pdf]

Identifying gene targets for brain-related traits using transcriptomic and methylomic data from blood

Qi et al.

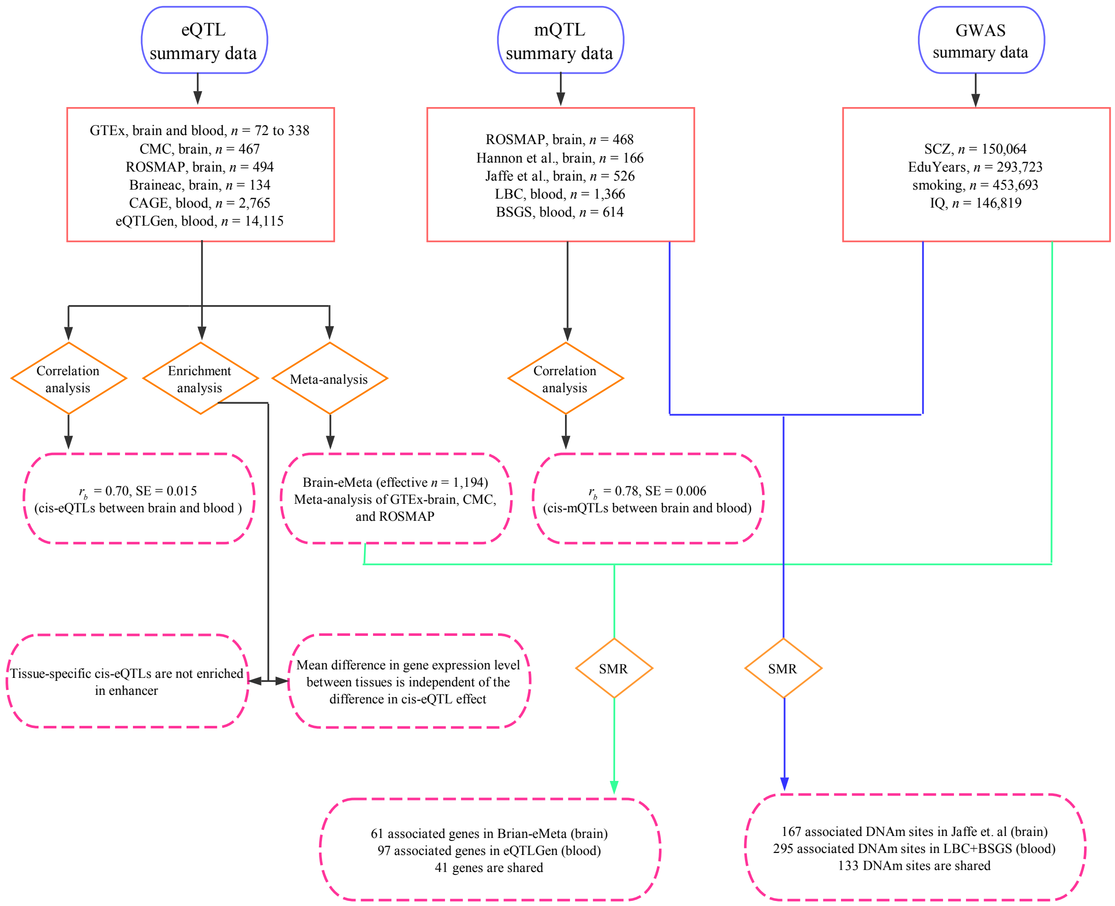

Supplementary Figure 1 Schematic overview of this study.

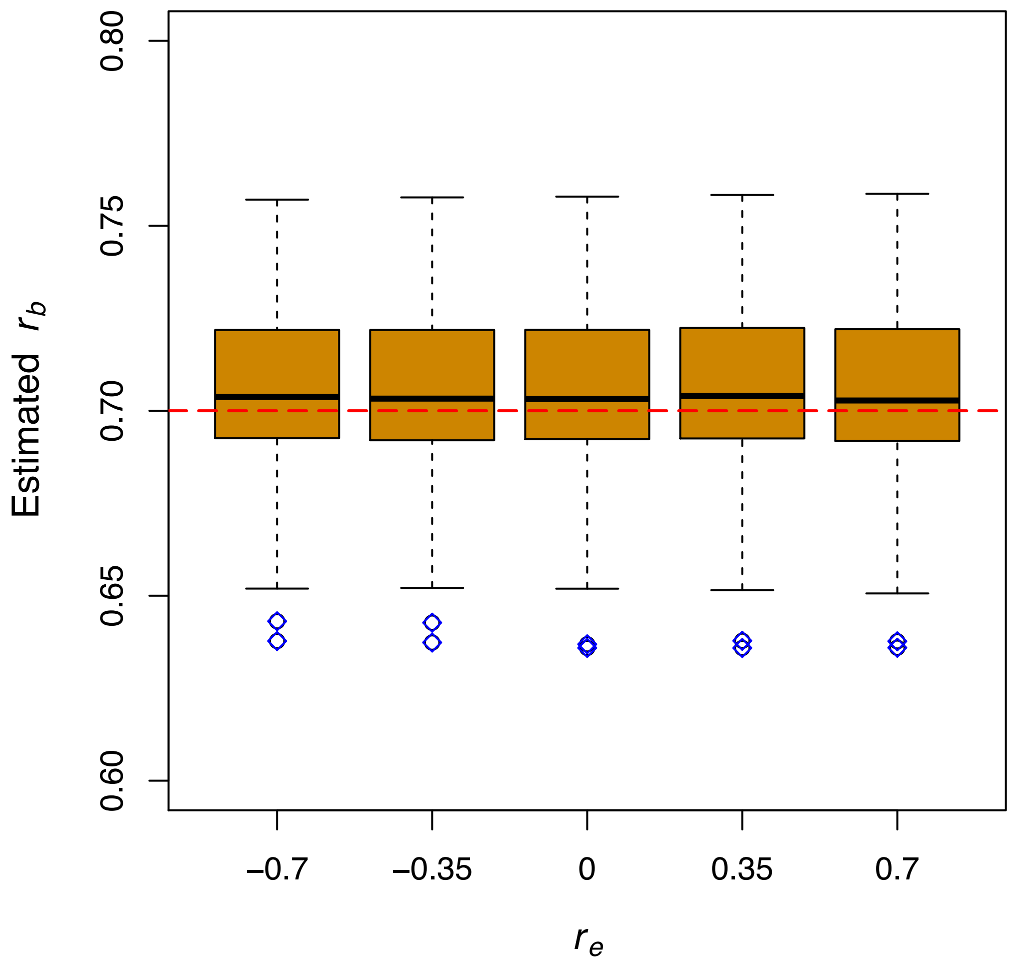

Supplementary Figure 2 Estimated r_b between two tissues at different levels of residual correlation (r_e) in simulations. The phenotypes were simulated based on the UK10K data set[1] with the SNPs in common with HapMap3 (see **Supplementary Note 1** for details). In brief, we simulated gene expression data in three tissues with correlated eQTL effects and residuals. In the r_b analysis of the simulated data, to avoid bias due to the winner's curse, we selected the top associated SNPs at $P_{eQTL} < 5\times10^{-8}$ in tissue #1, and estimated the correlation of top cis-eQTL effects between tissues #2 and #3. Each box in the figure represents the distribution of estimates from 100 simulation replicates. The red dash line represents the simulation parameter (i.e. ρ = 0.7). It is of note that here we compare the estimate of r_b between tissues #2 and #3 for genes with cis-eQTLs of relatively large effect (because of the ascertainment of the top cis-eQTLs by a stringent p-value threshold in tissue #1) with the parameter (ρ) used to simulate the correlation of cis-eQTLs effects between the tissues across all genes (**Supplementary Note 1**). Therefore, the estimate of r_b is not expected to be an unbiased estimator of ρ.

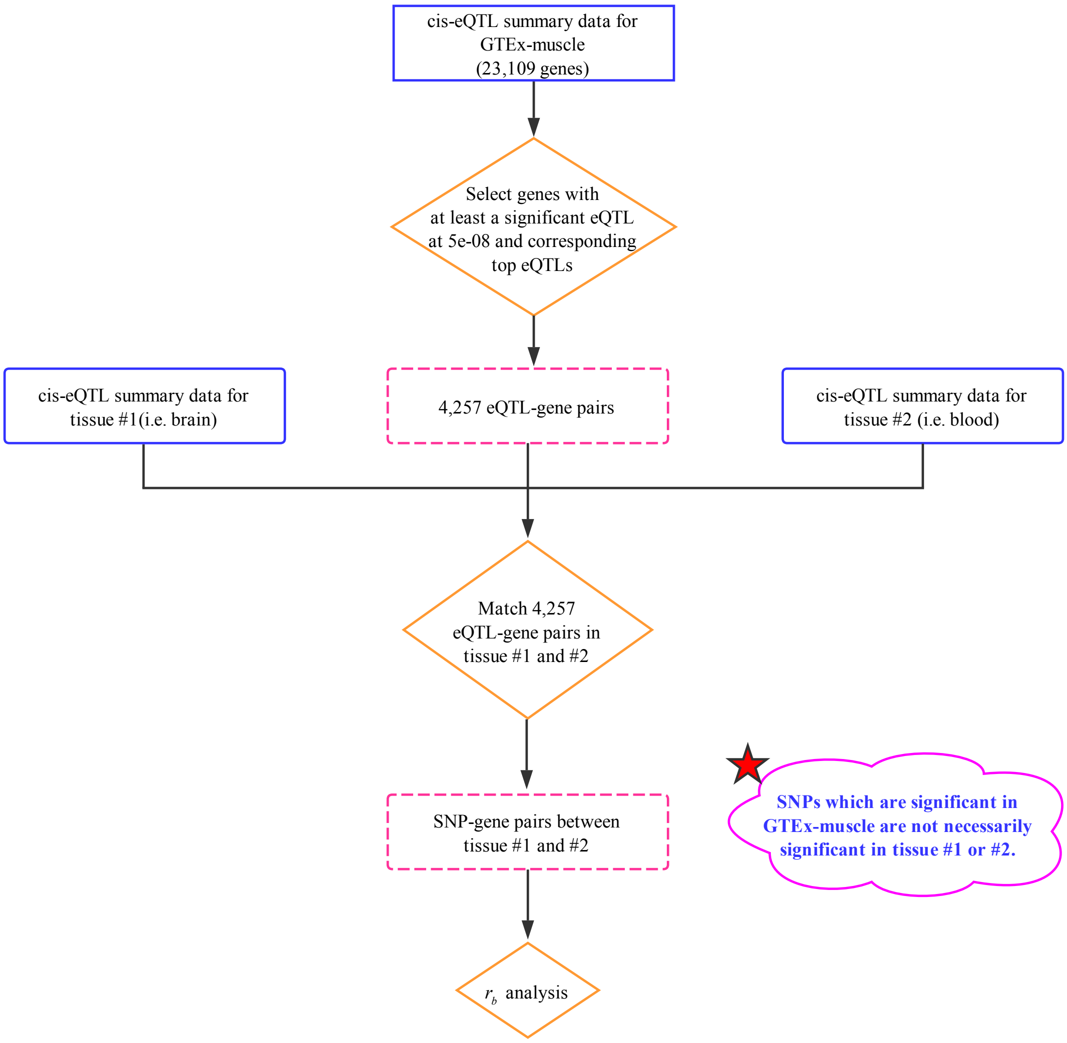

Supplementary Figure 3 Schematic overview of the r_b analysis.

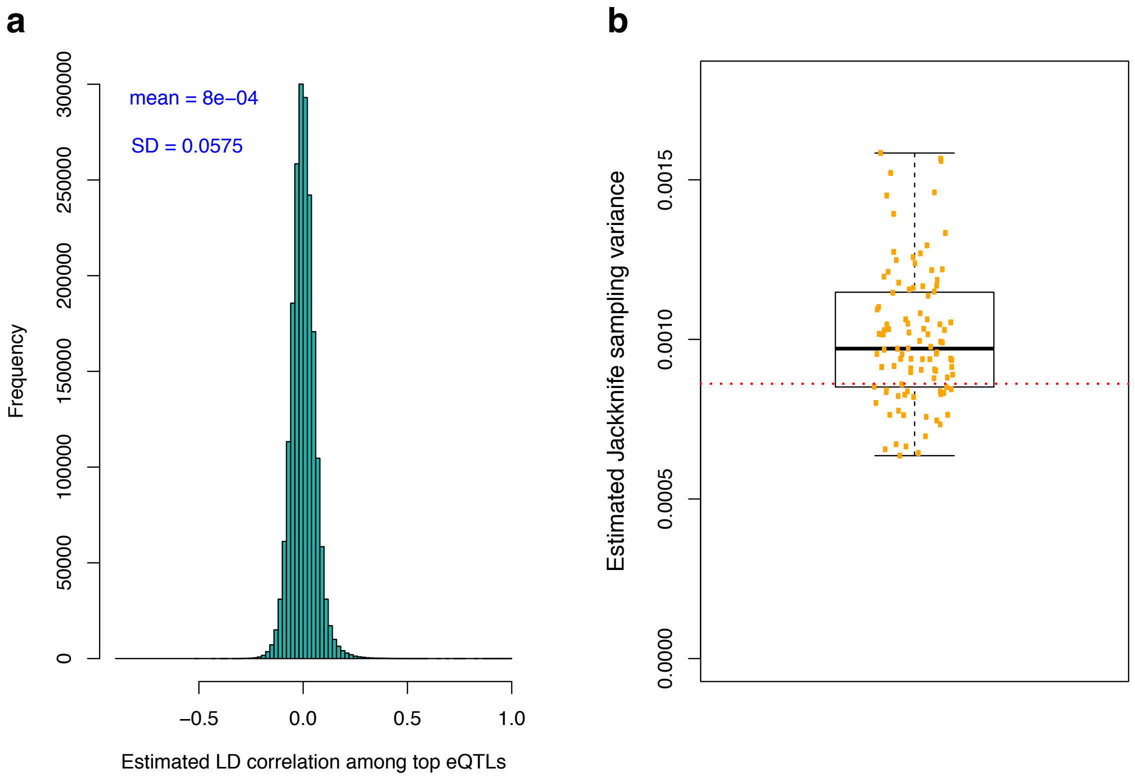

Supplementary Figure 4 Distributions of the LD correlations among 4,257 top cis-eQTLs and the estimated Jackknife sample variation from 100 simulation replicates. The 4,257 genes were selected at $P_{\text{eQTL}} < 5\times10^{-8}$ in GTEx-muscle. Shown in panel **a** is the distribution of the LD correlations among 4,257 top cis-eQTLs computed from the GTEx genotype data. The 4,257 cis-eQTLs are distributed across the whole genome with a mean LD r = 0.0008 (SD = 0.0575), suggesting that most of them are independent. Shown in panel **b** is the distribution of estimated Jackknife sample variance across 100 simulation replicates. We simulated gene expression data based on the UK10K data set[1] with the SNPs in common with HapMap3 (see **Supplementary Note 1** for details) in three tissues with correlated eQTL effects (r_b = 0.7) and residuals (r_e = -0.7). In the r_{b} analysis of the simulated data, to avoid bias due to the winner's curse, we selected the top associated SNPs at $P_{\text{eQTL}} < 5\times10^{-8}$ in tissue #1, and estimated the correlation of top cis-eQTL effects between tissues #2 and #3. The dots in panel **b** represent estimated Jackknife sample variance from 100 simulation replicates. The red dash line represents the variance of estimated r_b from 100 simulation replicates. It is of note that the mean Jackknife sample variance is slightly larger than the observed sample variance.

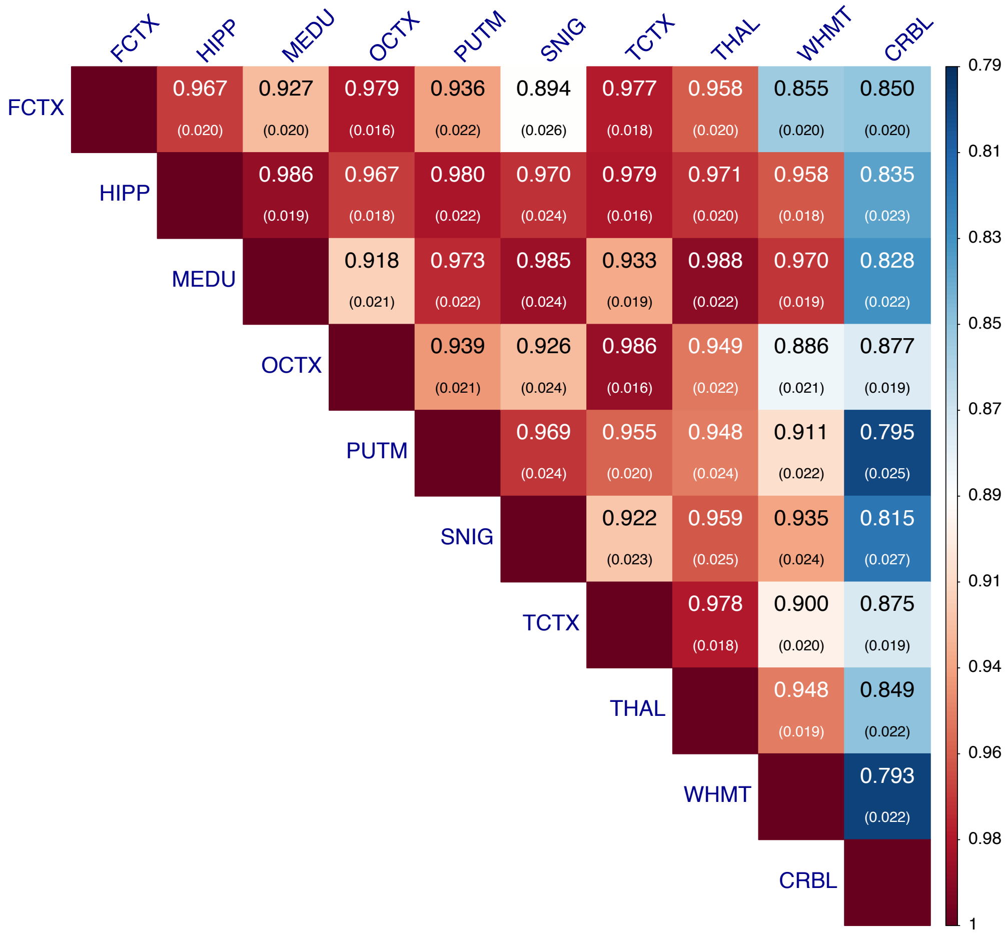

Supplementary Figure 5 Estimated r_b of cis-eQTLs among 10 brain regions in Braineac. The top cis-eQTLs were selected from GTEx-muscle at $P_{\text{eQTL}} < 5\times10^{-8}$. We matched the Braineac data with GTEx-muscle by gene symbols and excluded genes tagged by multiple probes. Shown in each cell is the estimate of r_b with its standard error given in the parentheses (**Methods**). FCTX, frontal cortex; HIPP, hippocampus; MEDU, medulla (specifically inferior olivary nucleus); OCTX, occipital cortex (specifically primary visual cortex); PUTM, putamen; SNIG, substantia nigra; THAL, thalamus; TCTX, temporal cortex; WHMT, intralobular white matter; CRBL, cerebellar cortex.

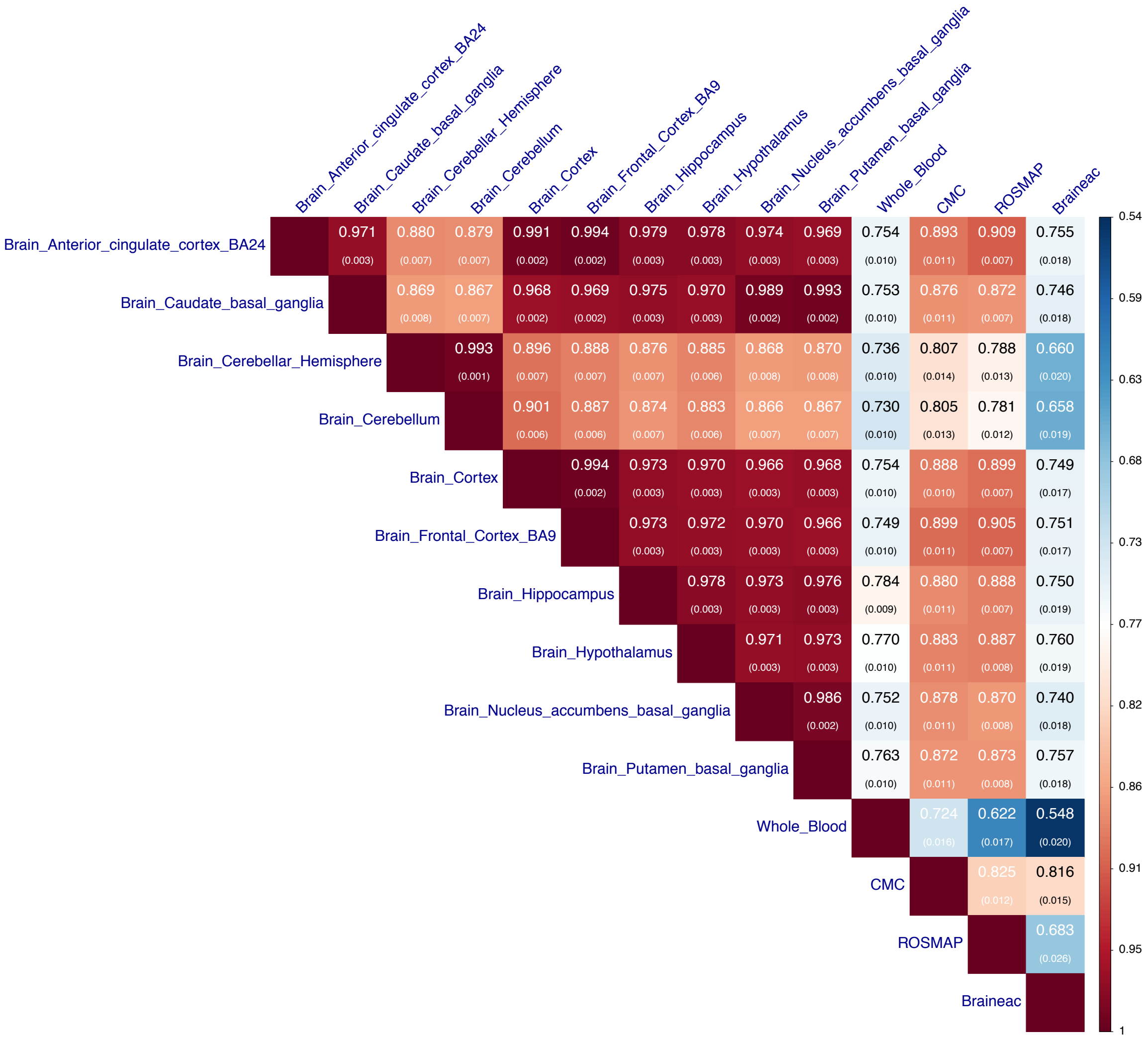

Supplementary Figure 6 Estimated r_b of the scaled cis-eQTL effects between brain regions, between brain and blood tissues, and between data sets. We know that the SE of an estimated eQTL effect is a function of the minor allele frequency (MAF) of the eQTL. In the analysis presented in **Figure 1**, we used the mean SE squared across genes to estimate the variance of estimation errors (**Methods**). However, MAFs of cis-eQTLs are different across genes. We therefore scaled the eQTL effect size and SE as $\hat{b}_{scale} = \hat{b}\sqrt{2p(1-p)}$ and $s_{scale} = s\sqrt{2p(1-p)}$, where $\hat{b}_{scale}$ is interpreted as the eQTL effect size per-standardized genotype, p is MAF, $\hat{b}$ is the estimated eQTL effect, and s is the standard error of $\hat{b}$. We then re-ran the r_b analysis using the scaled cis-eQTL effects and SEs (**Methods**). The top cis-eQTLs were selected from the GTEx-muscle data at $P_{\text{eQTL}} < 5\times10^{-8}$. Shown in each cell is the estimate of r_b with its standard error given in the parentheses. These results are almost identical to those presented in **Figure 1**, suggesting that the method is robust to scale transformation of the eQTL effects.

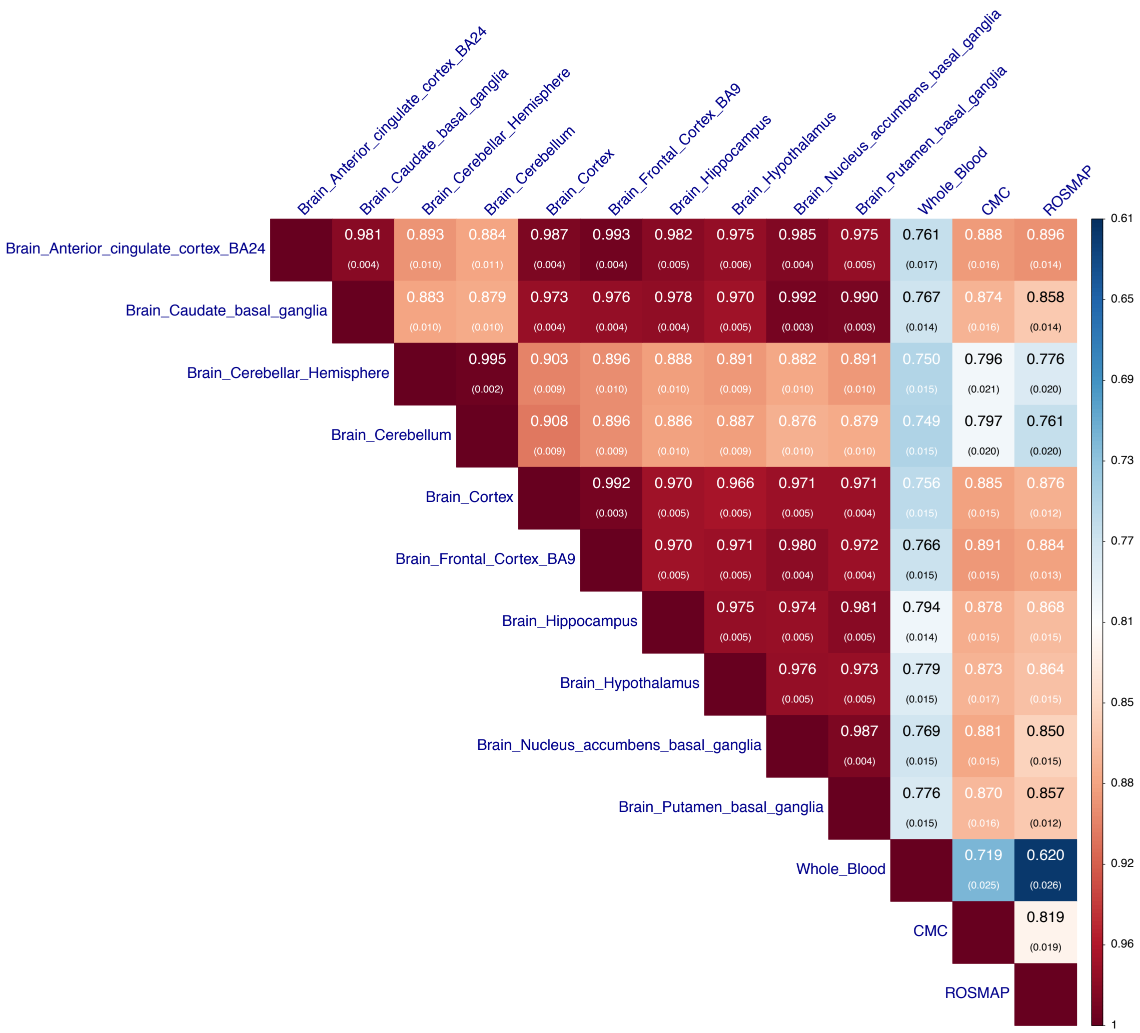

Supplementary Figure 7 Estimated r_b of cis-eQTLs between brain regions, between brain and blood tissues, and between data sets, excluding the cis-QTLs within 10Kb of the promoter regions. Shown in each cell is the estimate of r_b with its standard error given in the parentheses (**Methods**).

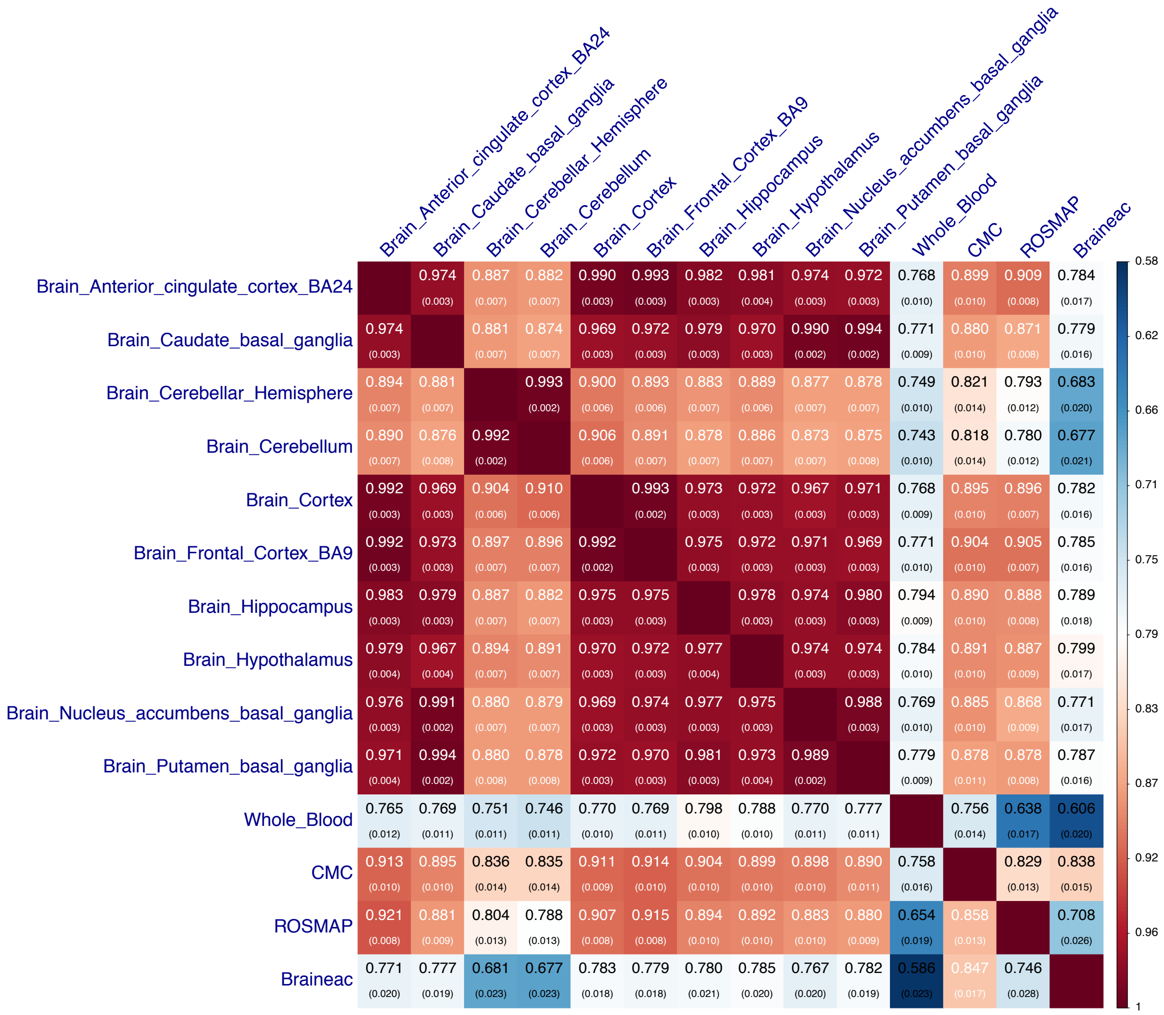

Supplementary Figure 8 Estimated r_b of cis-eQTLs between brain regions, between brain and blood tissues, and between data sets, excluding the housekeeping (HK) genes obtained from Lin et al.[2] (upper right) and Eisenberg et al.[3] (lower left). There were 4,257 genes in our ascertained gene list. The number of HK genes in the ascertained gene list (e.g. m = 220 for Lin et al.) is significantly higher than what we would expect from a random sample of genes (mean = 187 with SD = 12.2 from 2,000 random gene sets). This is expected, because HK genes are defined as a set of genes expressed across most cell types and tissues, which are expected to be enriched in genes expressed in both brain and blood. We re-ran the r_b analysis excluding the HK genes. The results are almost identical to those presented in **Figure 1**, suggesting that the estimates of r_b are robust to the inclusion/exclusion of HK genes. Shown in each cell is the estimate of r_b with its standard error given in the parentheses (**Methods**).

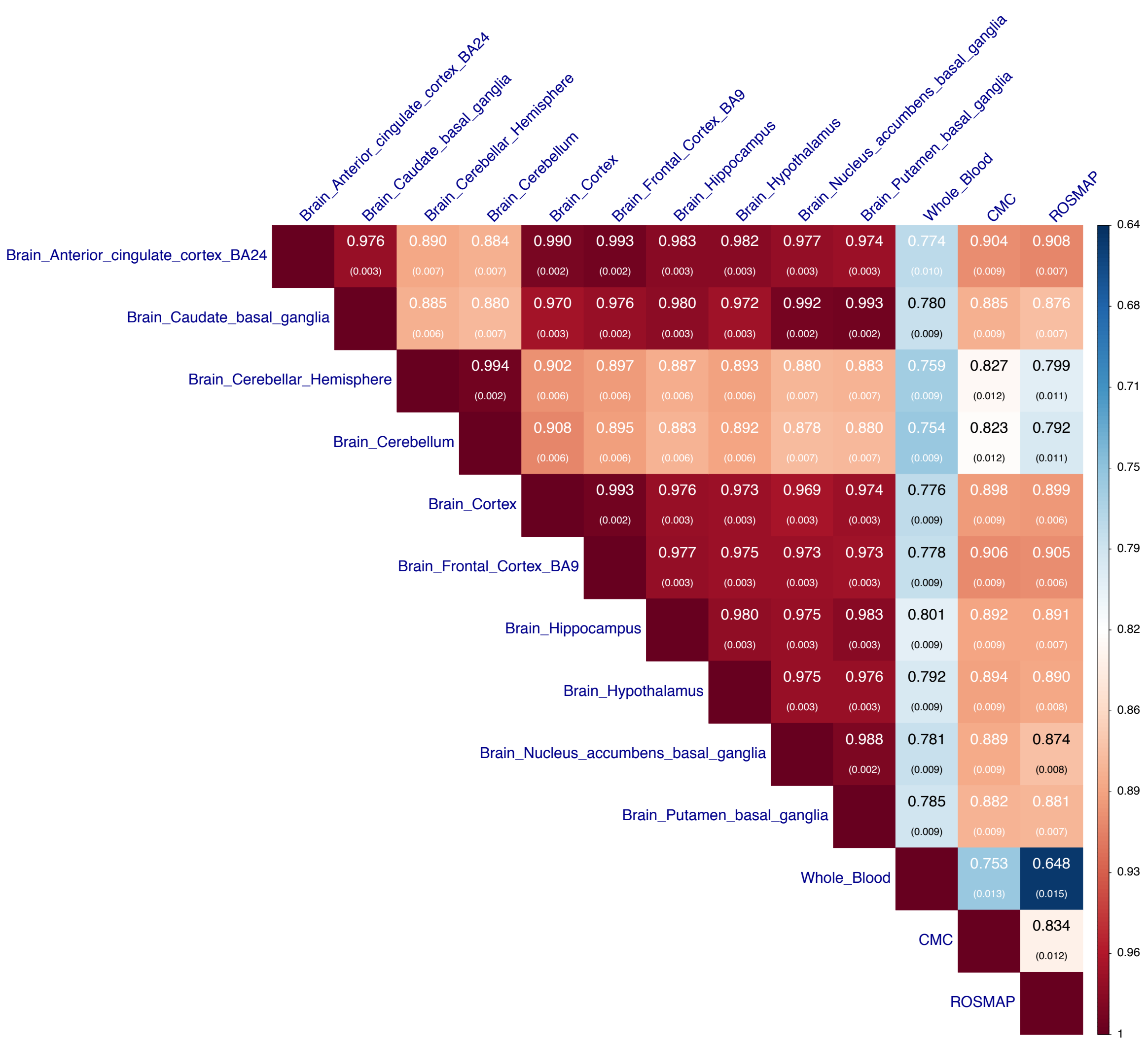

Supplementary Figure 9 Estimated r_b of cis-eQTLs including both the primary and secondary signals between brain regions, between brain and blood tissues, and between data sets. Conditional analysis was performed in each of the cis-eQTL regions in GTEx-muscle using a summary-data-based conditional analysis method in GCTA[4,5]. We identified secondary signals by the conditional analysis for 659 probes. Shown in each cell is the estimate of r_b with its standard error given in the parentheses (**Methods**).

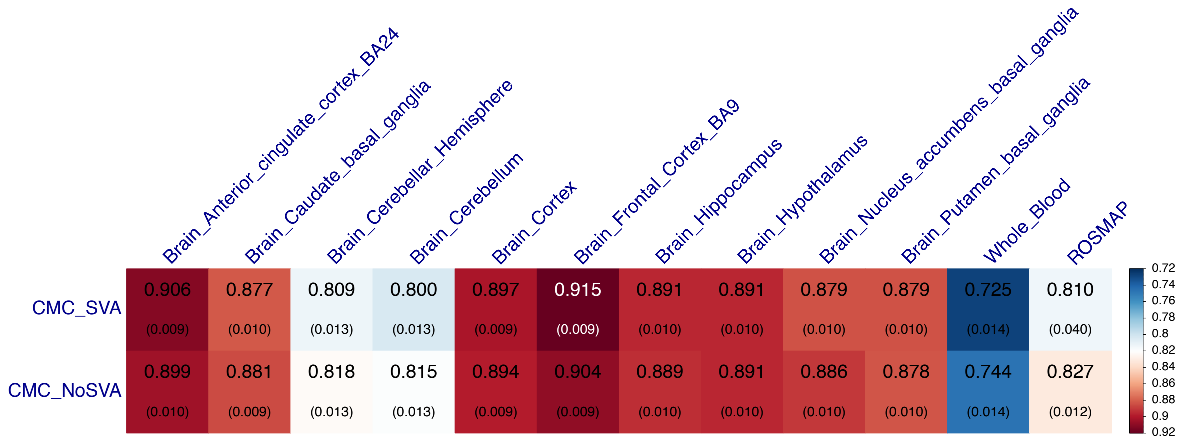

Supplementary Figure 10 Estimated r_b of cis-eQTLs between two versions of CMC data and GTEx-brain, GTEx-blood and ROSMAP. CMC_SVA represents gene expression data in CMC adjusted by the surrogate variable analysis (SVA), where SVA is an approach used to overcome the problems caused by heterogeneity in expression studies[6]. CMC_NoVSA represents CMC data without SVA adjustment. Shown in each cell is the estimate of r_{b} with its standard error given in the parentheses (**Methods**).

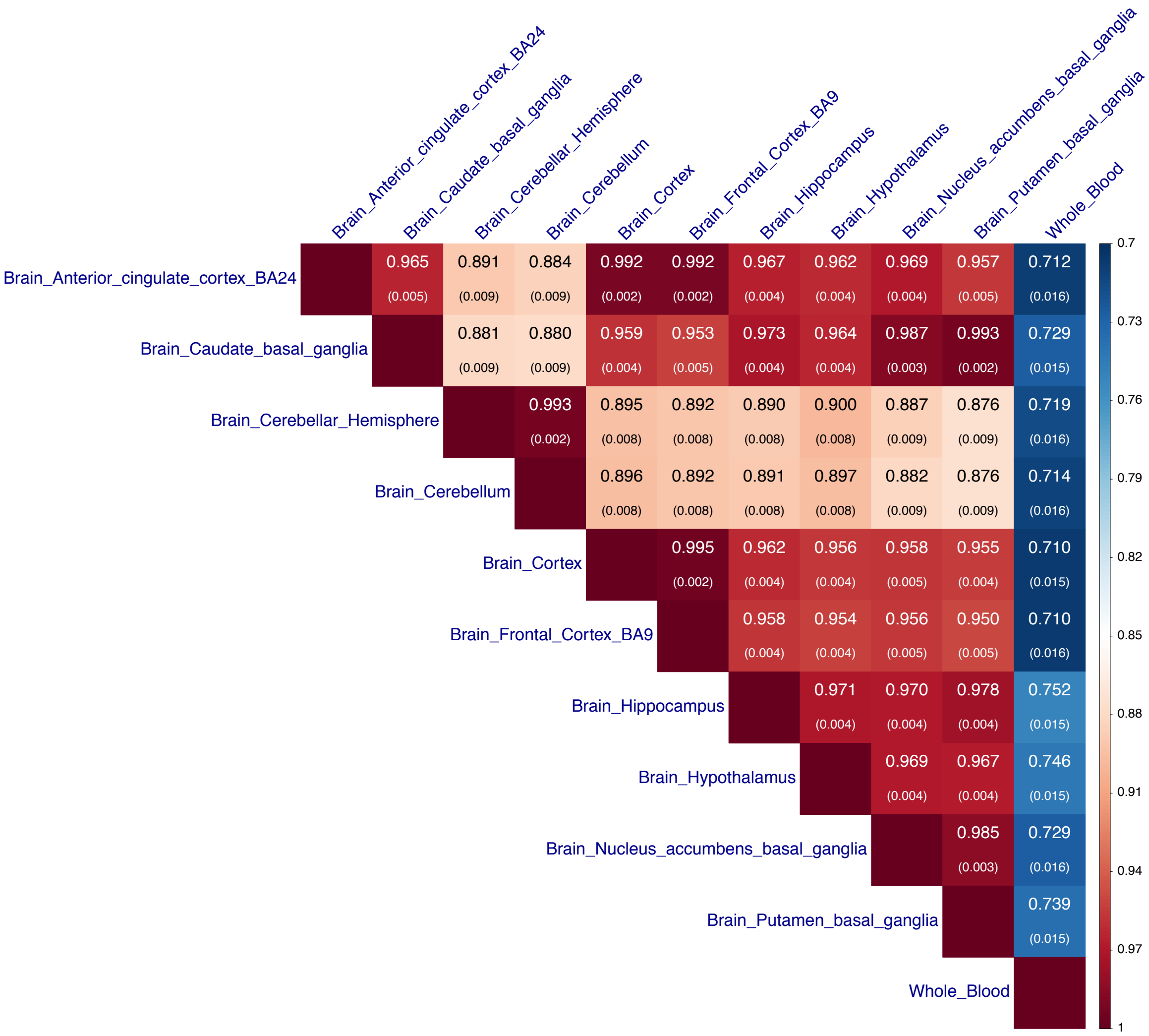

Supplementary Figure 11 Estimated r_b among 11 GTEx tissues for cis-eQTL ascertained from CMC. The top cis-eQTLs were selected from the CMC data at $P_{\text{eQTL}} < 5\times10^{-8}$. Shown in each cell is the estimate of r_b with its standard error given in the parentheses (**Methods**).

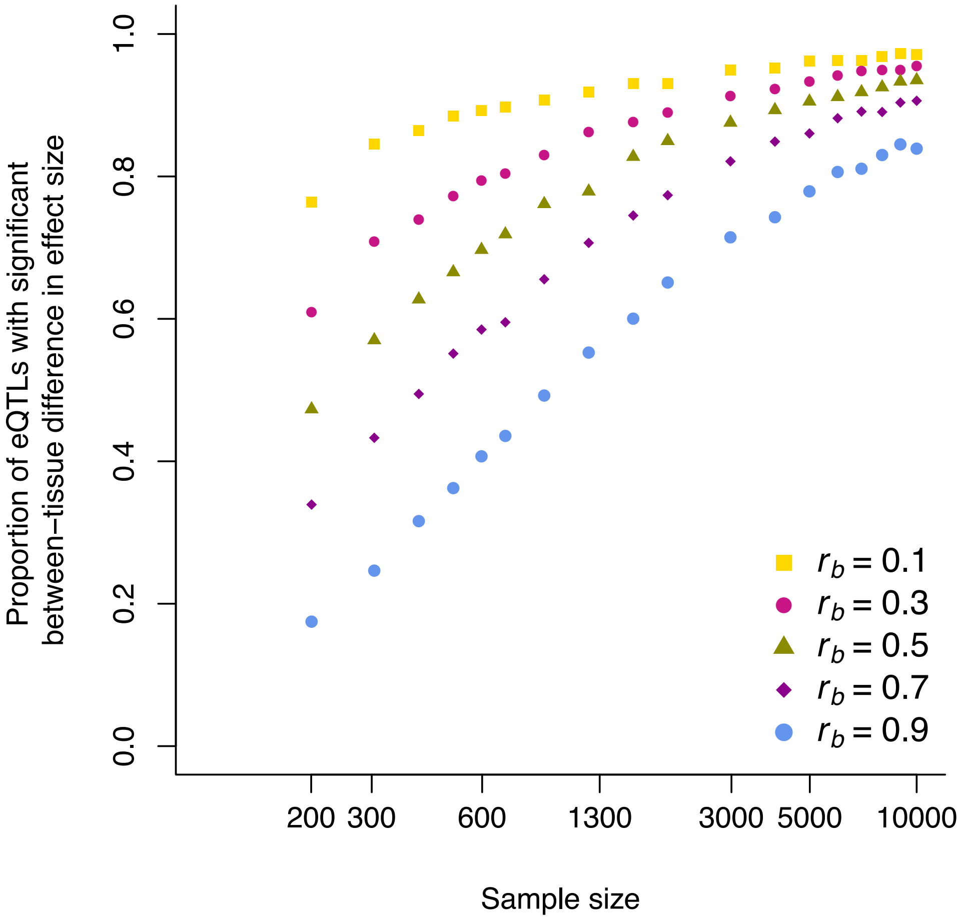

Supplementary Figure 12 Proportion of eQTLs with significant difference in effect size between-tissues (after Bonferroni correction for multiple testing) as a function of sample size and r_b. Each dot represents the mean estimate from 1,000 simulation replicates (**Supplementary Note 1**).

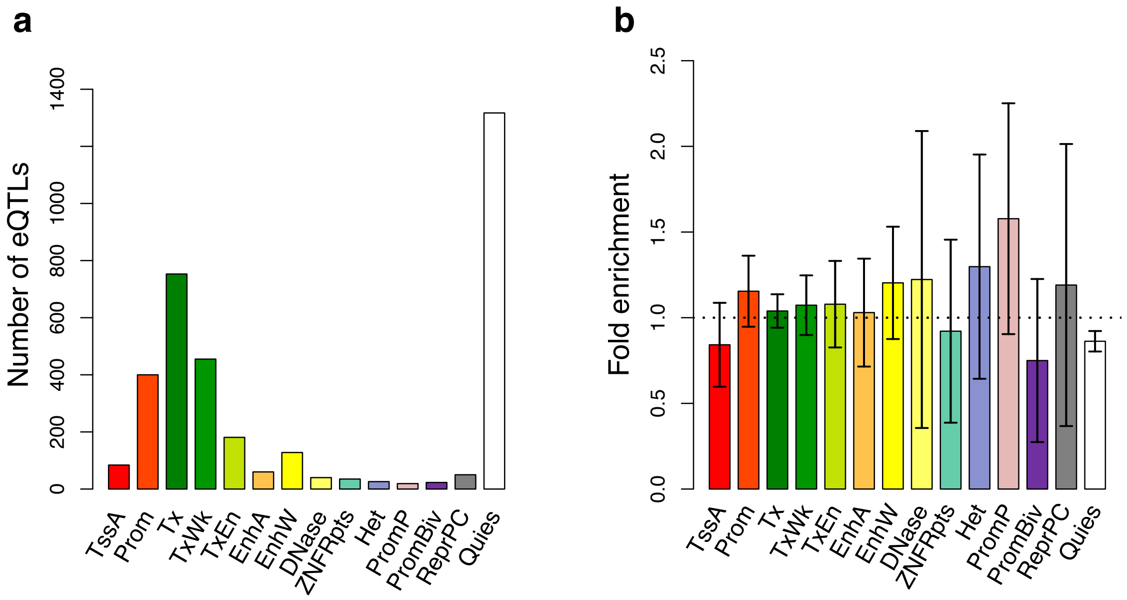

Supplementary Figure 13 Enrichment of cis-eQTLs with tissue-specific effects in functional annotations. **a** The distribution of cis-eQTLs across 14 functional categories derived from Roadmap Epigenomics Mapping Consortium (REMC) (**Methods**). **b** Estimated enrichment of test-statistics for the difference (T_D) (testing for the difference in cis-eQTL effect between GTEx-cerebellum and GTEx-blood) in each functional category (**Methods**). Error bars represent 95% confidence intervals around the estimates. The black dash line represents fold enrichment of 1. Different colors in panels **a** and **b** represent the 14 functional categories from REMC: TssA, active transcription start site; Prom, upstream/downstream TSS promoter; Tx, actively transcribed state; TxWk, weak transcription; TxEn, transcribed and regulatory Prom/Enh; EnhA, active enhancer; EnhW, weak enhancer; DNase, primary DNase; ZNF/Rpts, state associated with zinc finger protein genes; Het, constitutive heterochromatin; PromP, Poised promoter; PromBiv, bivalent regulatory states; ReprPC, repressed Polycomb states; and Quies, a quiescent state.

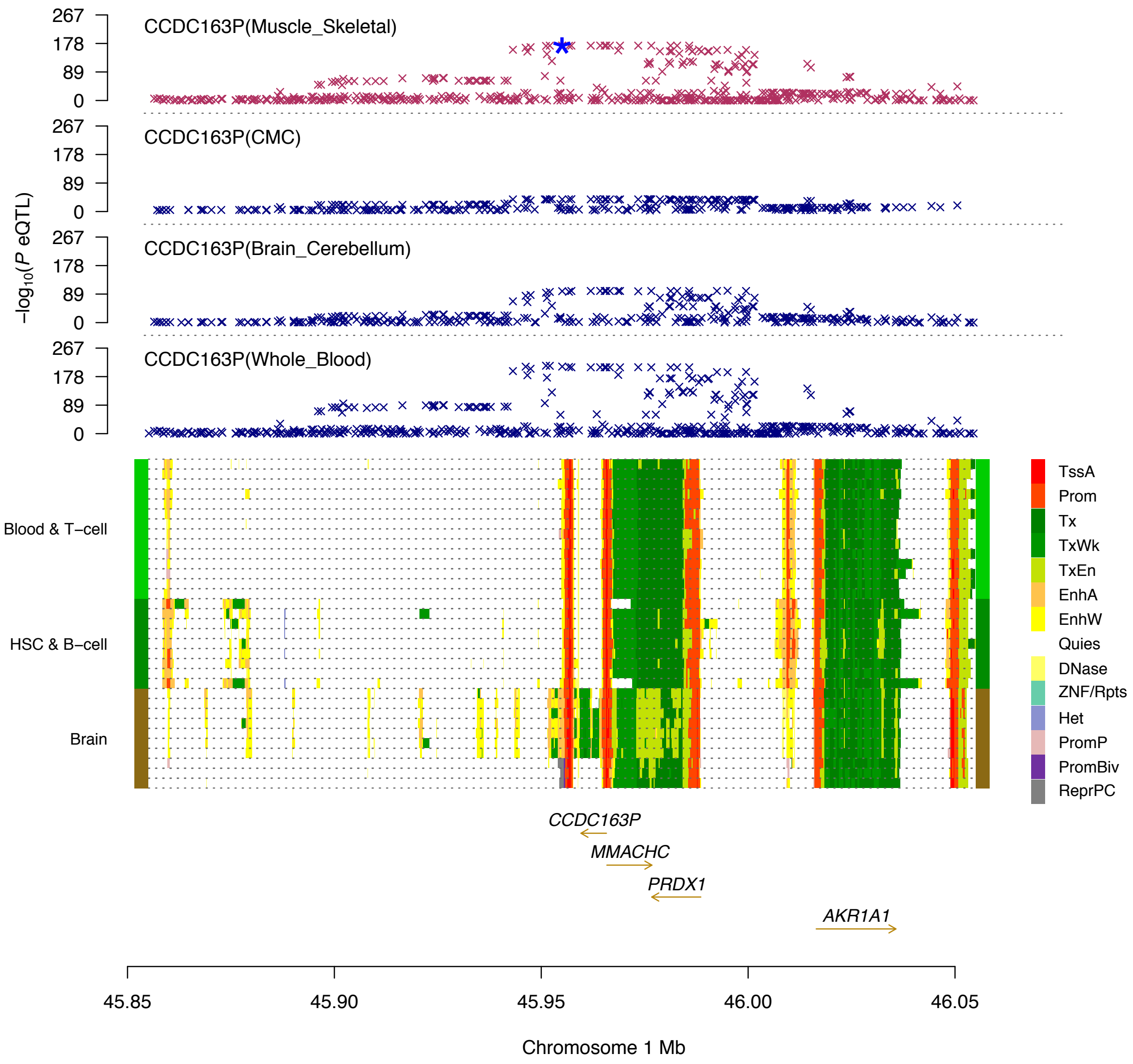

Supplementary Figure 14 Shown is an example where a top cis-eQTL with tissue-specific effect is located in an enhancer region. The top eQTL was selected from GTEx-muscle at $P_{eQTL} < 5\times10^{-8}$. Showing in the top four plots are $-\log_{10}(P$ values) for eQTLs of all the cis-SNPs for gene CCDC163P in GTEx-muscle, CMC, GTEx-cerebellum, and GTEx-blood respectively. Each row represents a REMC sample. The blue asterisk in the top plot indicates the top eQTL which co-localizes a tissue-specific enhancer region in brain. The bottom plot shows 14 chromatin state annotations (indicated by different colours) of the region derived from the Roadmap Epigenomics Mapping Consortium (REMC) (**Methods**). TssA, active transcription start site; Prom, upstream/downstream TSS promoter; Tx, actively transcribed state; TxWk, weak transcription; TxEn, transcribed and regulatory Prom/Enh; EnhA, active enhancer; EnhW, weak enhancer; DNase, primary DNase; ZNF/Rpts, state associated with zinc finger protein genes; Het, constitutive heterochromatin; PromP, Poised promoter; PromBiv, bivalent regulatory states; ReprPC, repressed Polycomb states; and Quies, a quiescent state.

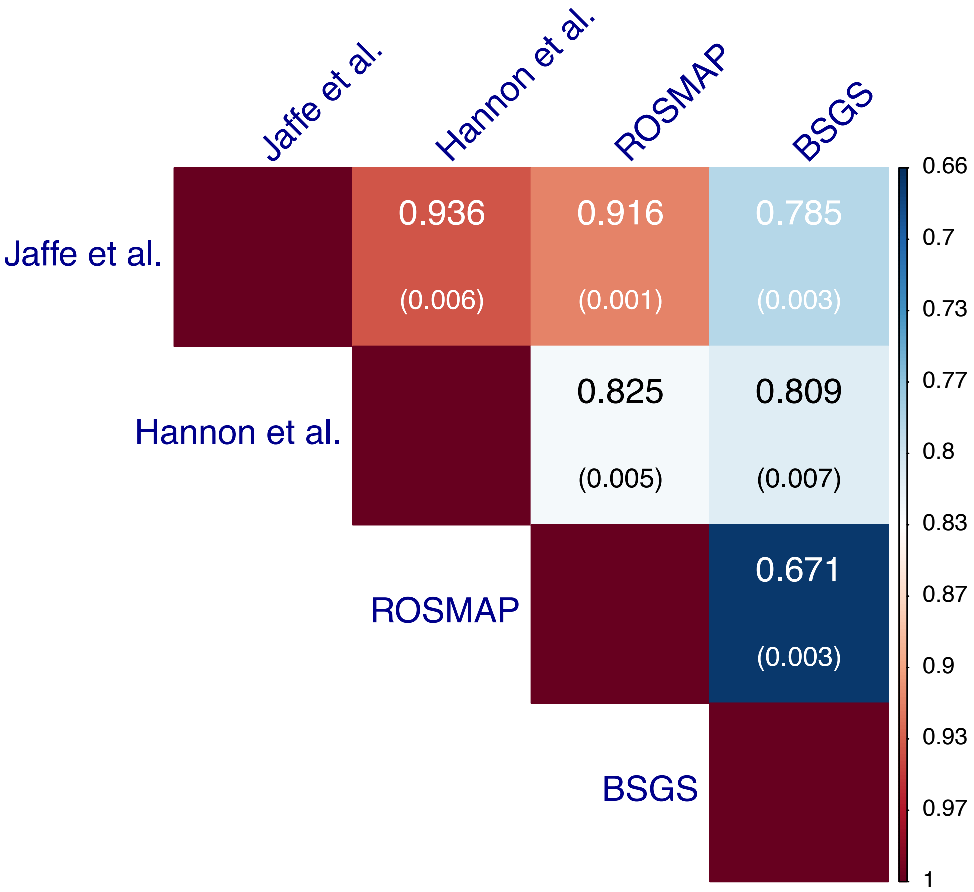

Supplementary Figure 15 Estimated r_b of cis-mQTLs between brain and blood in different samples. The top cis-mQTLs were ascertained in LBC at $P_{\mathrm{mQTL}} < 5\times10^{-8}$. In the ROSMAP data, only SNPs within 5Kb of the DNAm probes were available which might result in a downward bias of the r_{b} estimate.

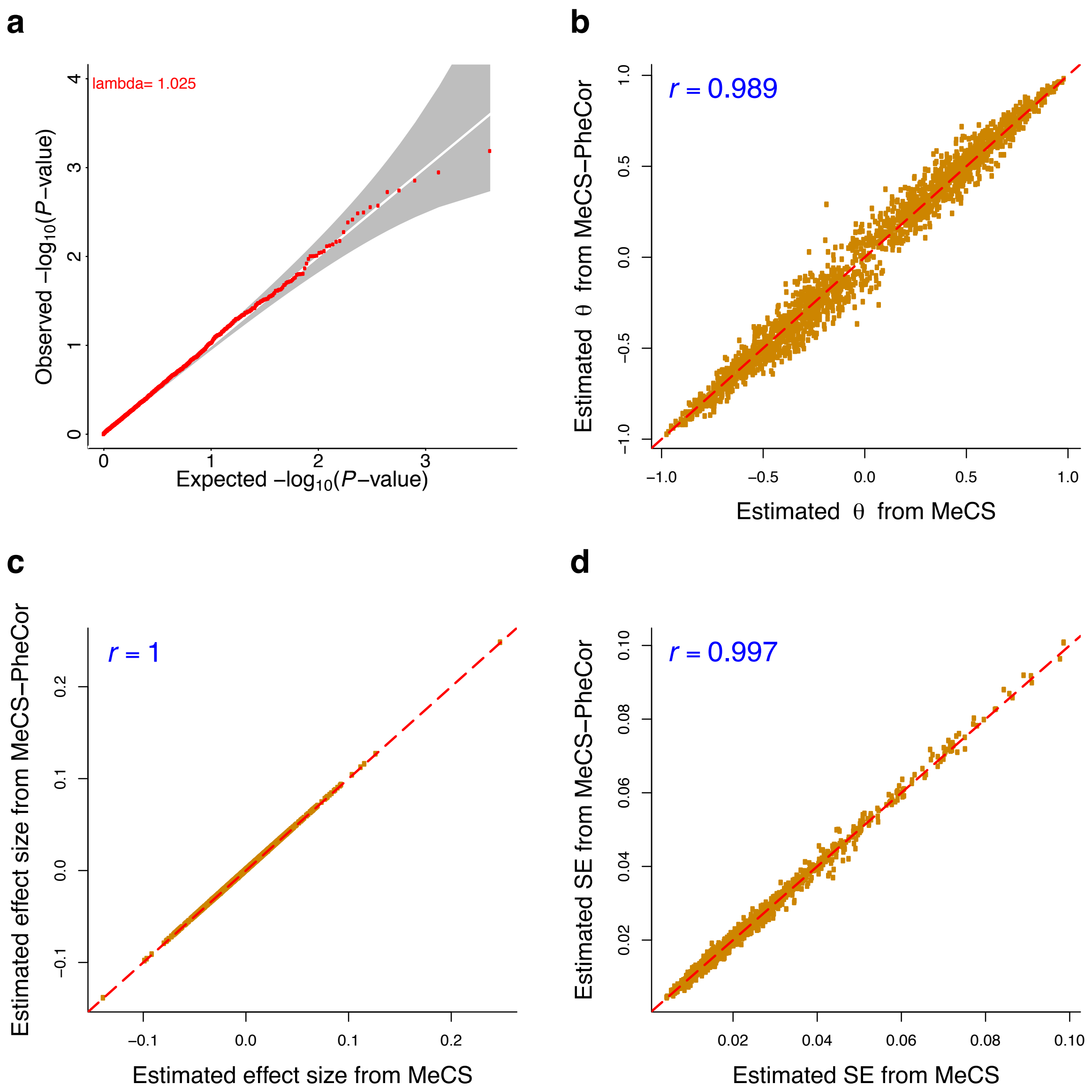

Supplementary Figure 16 MeCS results from simulations under the null hypothesis that there is no cis-eQTL effect. MeCS-PheCor represents a MeCS analysis where correlation of estimation error (θ) is estimated by phenotypic correlation from individual-level data. **a** Quantile-quantile plot for MeCS under the null model. **b** Estimated θ from summary data vs. that from individual-level data (sample overlap = 1). **c** Estimated effect size from MeCS vs. that from MeCS-PheCor. **d** Estimated SE from MeCS vs. that from MeCS-PheCor. Red dash lines in panel **b**, **c**, and **d** represent the diagonal lines ($y = x$).

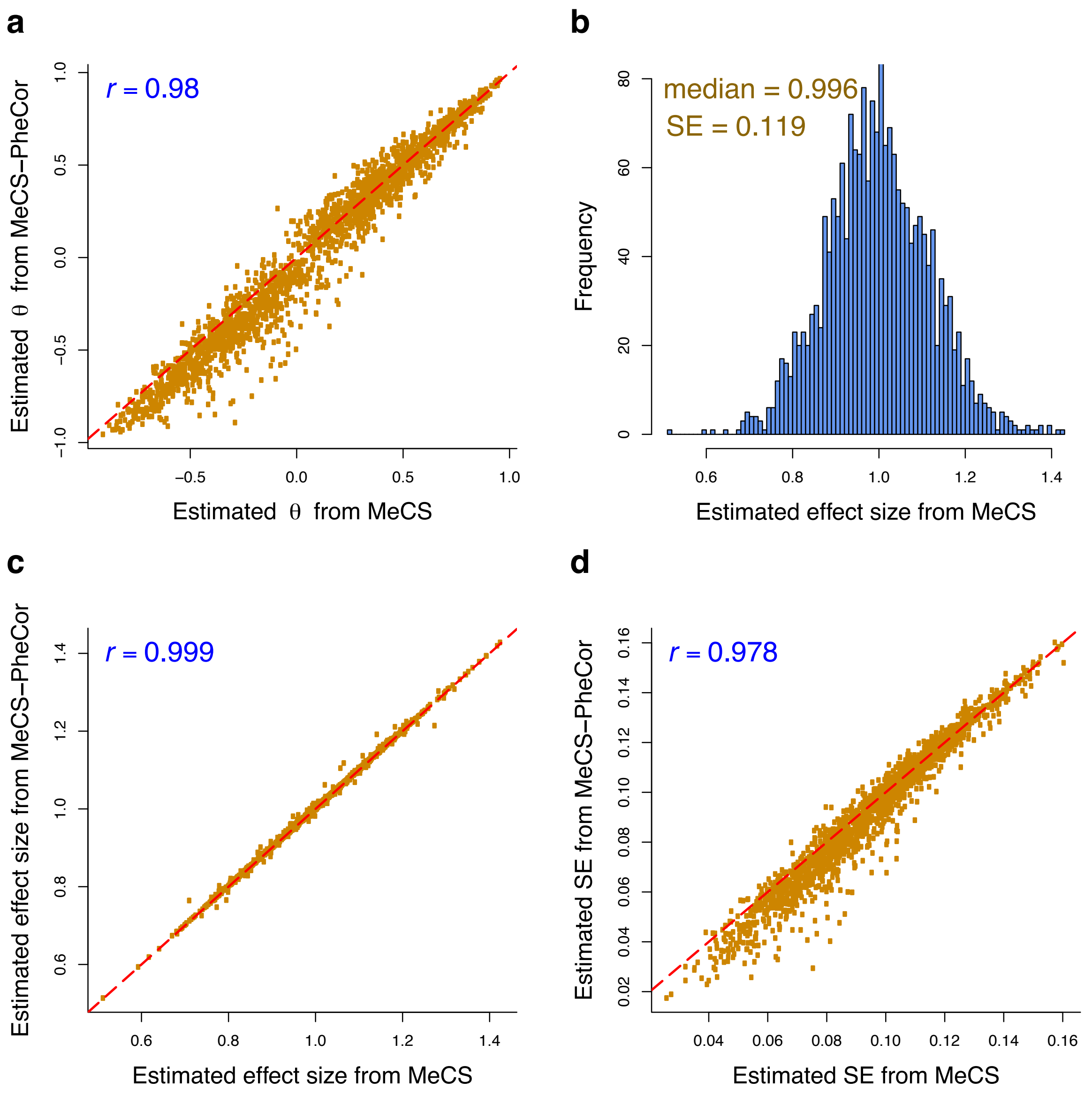

Supplementary Figure 17 MeCS results from simulations under the alternative hypothesis that the eQTL effects are non-zero and vary across tissues. MeCS-PheCor represents a MeCS analysis where θ is estimated from individual-level data. **a** Estimated θ from summary-level data vs. that from individual-level data. **b** Distribution of estimated meta-analysis effect size from MeCS. **c** Estimated meta-analysis effect size from MeCS vs. that from MeCS-PheCor. **d** Estimated SE from MeCS vs. that from MeCS-PheCor. Red dash lines in panel **a**, **c**, and **d** represent the diagonal lines ($y = x$).

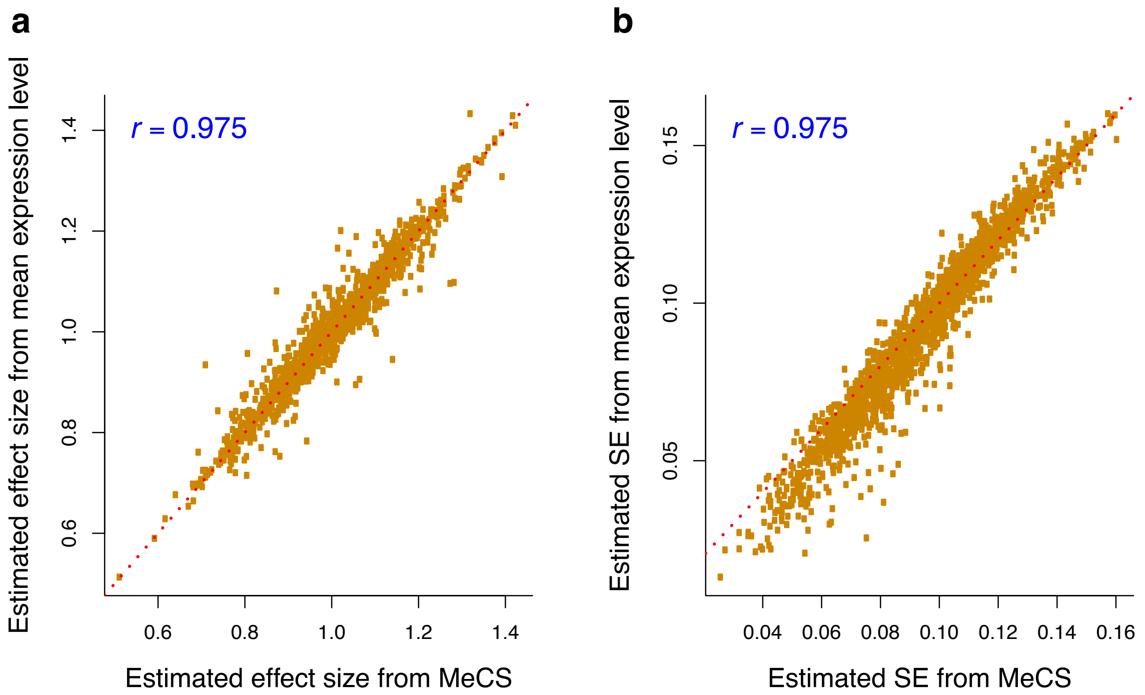

Supplementary Figure 18 Estimates of eQTL effects and SE from MeCS vs. those from univariate analysis of mean expression level. In the analysis of mean expression level, we performed a standard GWAS analysis of the mean gene expression level of two tissues. **a** Meta-analysis effect size from MeCS vs. that from a univariate analysis of mean expression level. **b** Estimated SE from MeCS vs. that from a univariate analysis of mean expression level. Red dash lines represent the diagonal lines ($y = x$).

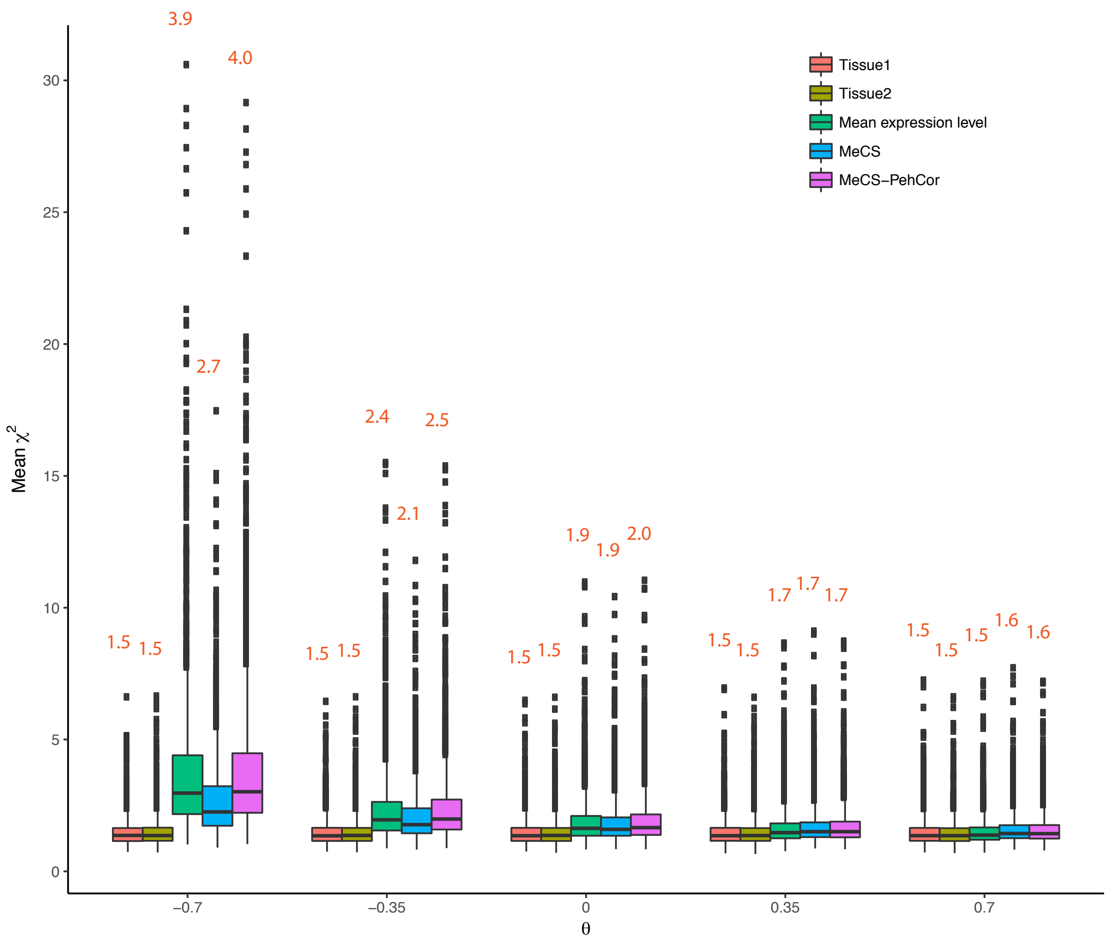

Supplementary Figure 19 Mean χ^2 across all eQTLs under different levels of θ (-0.70, -0.35, 0, 0.35 and 0.70). Each column is a box-plot of the mean χ^2 values from 1,000 simulation replicates under different levels of θ. The mean value of each column is labelled in red. Tissue 1 and Tissue 2: single-tissue analyses. Mean expression level: a univariate analysis of the mean expression level of two tissues. MeCS-PheCor: MeCS analysis with θ estimated from individual-level data (sample overlap = 1).

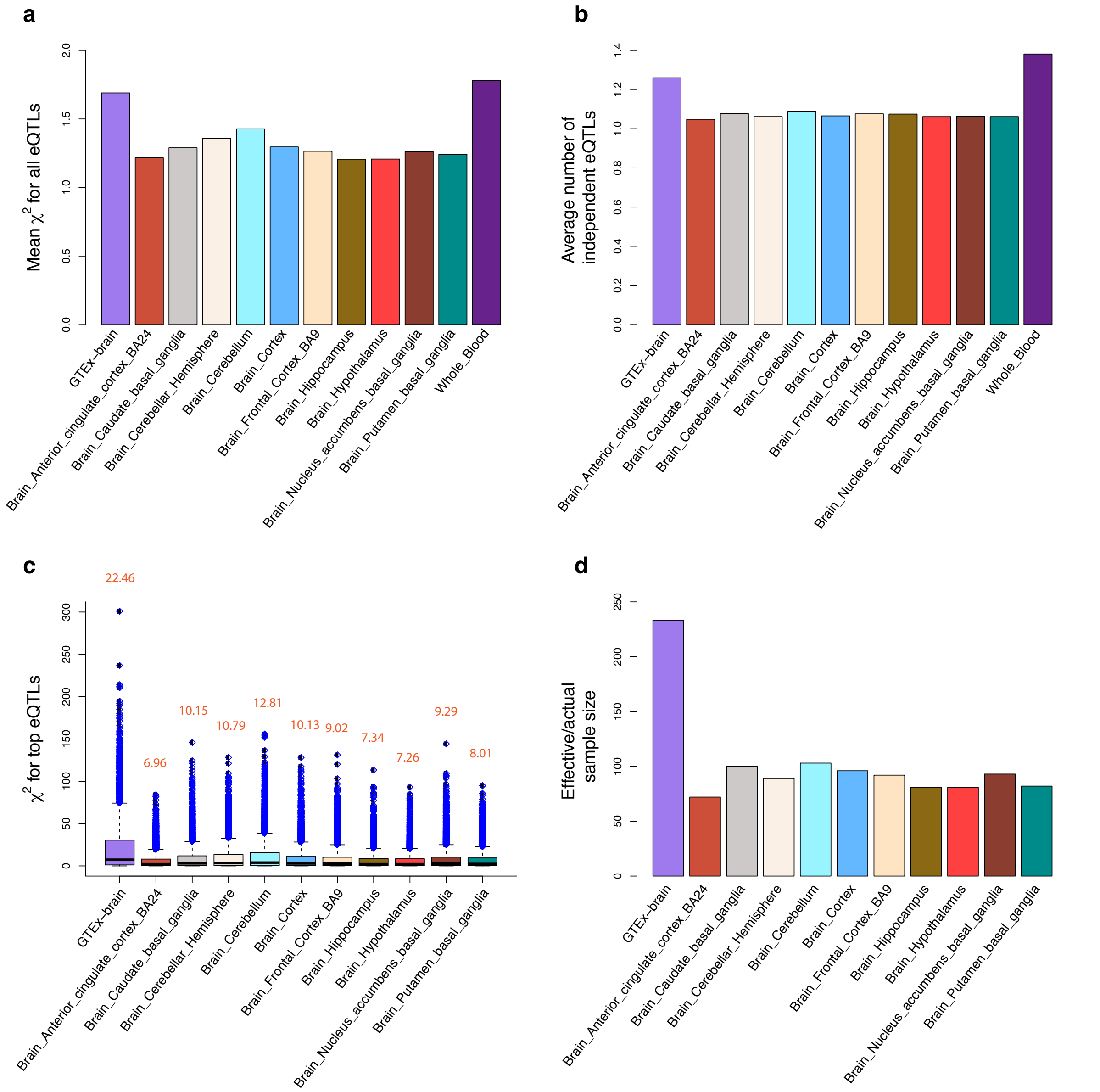

Supplementary Figure 20 MeCS analysis of the 10 GTEx brain regions. **a** Average mean χ^2 for all eQTLs across all probes from GTEx-brain, 10 brain regions individually, and GTEx-blood. Note that GTEx-brain represents a MeCS analysis of 10 GTEx brain regions. **b** Average number of independent significant eQTLs (from PLINK clumping analysis) per gene in GTEx-brain, 10 brain regions individually, and GTEx-blood. **c** Box-plots of the χ^2 values of the top cis-eQTLs in GTEx-brain and 10 GTEx brain regions where the top cis-eQTLs were ascertained in GTEx-blood at $P < 5\times10^{-8}$. The mean value of each column is labelled in red. **d** Effective/actual sample size for GTEx-brain and 10 brain regions.

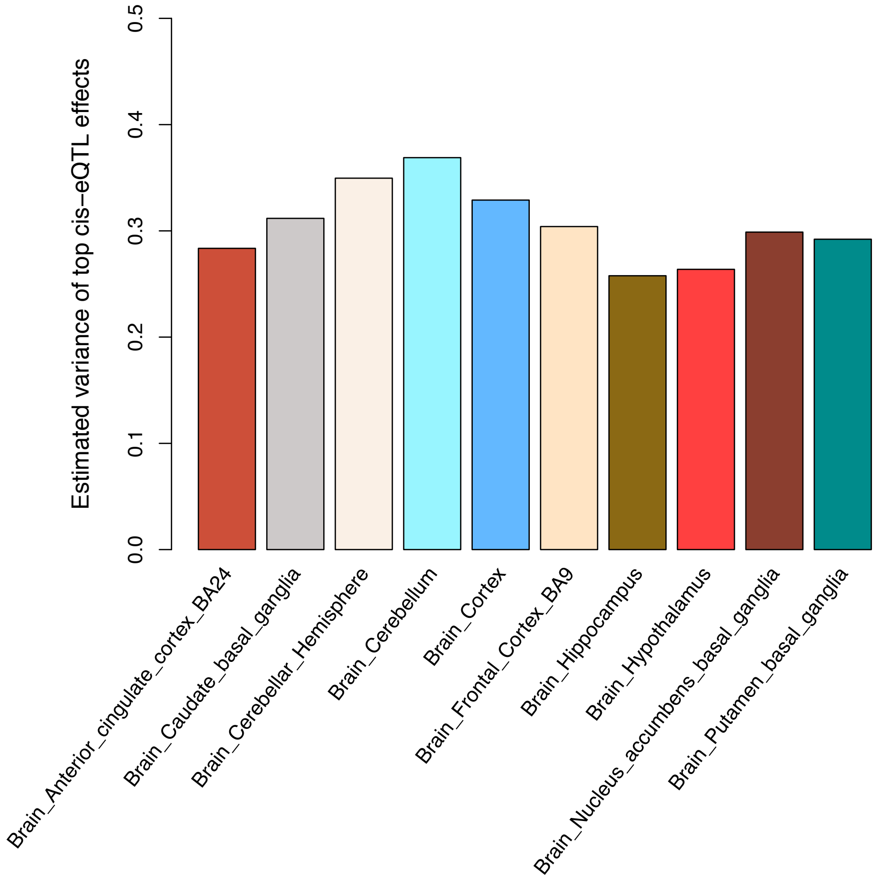

Supplementary Figure 21 Estimated variance of top cis-eQTL effects across genes in each brain region in GTEx. The top cis-eQTLs for 4,257 genes (a cis-eQTL per gene) were selected at $P < 5\times10^{-8}$ in GTEx-muscle. The variance of SNP effects was estimated by an approximate approach $\widehat{\text{var}}(b) \approx \widehat{\text{var}}(\hat{b}) - \widehat{\text{mean}}(\text{SE}^2)$ where $\widehat{\text{var}}$ and $\widehat{\text{mean}}$ denote the sample variance and mean across genes, respectively.

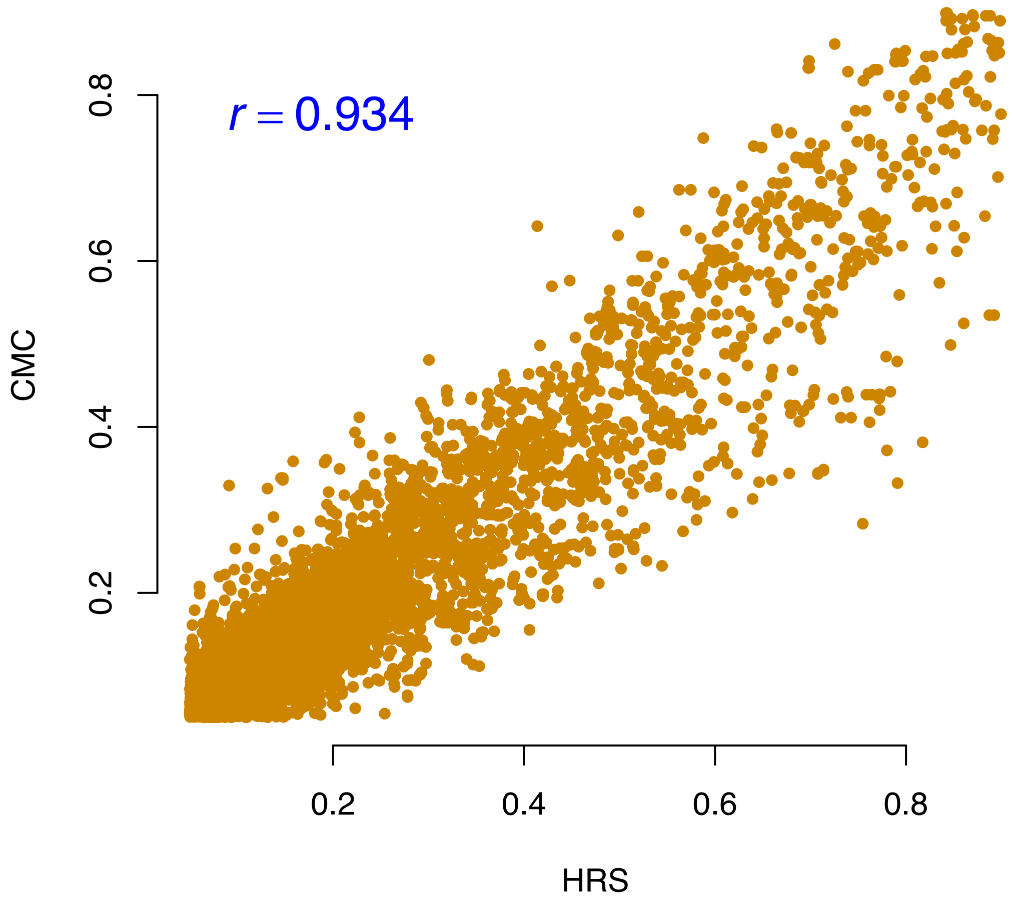

Supplementary Figure 22 Relationship of LD r^2 between CMC and the Health and Retirement Study (HRS). HRS is used as the reference sample in HEIDI test for LD estimation. Shown are the LD r^2 between 1,500 pairs of adjacent common SNPs on chromosome 22 estimated in the CMC (n = 621) and HRS data (n = 7,703 European Americans). Both x- and y-axes are limited to the range between 0.05 and 0.9 because the HEIDI test only uses LD within this range. There are observable differences in LD due to sampling because of finite sample sizes. These differences might lead to an increased rejection rate for HEIDI but not affecting the false discovery rate of the SMR & HEIDI analysis.

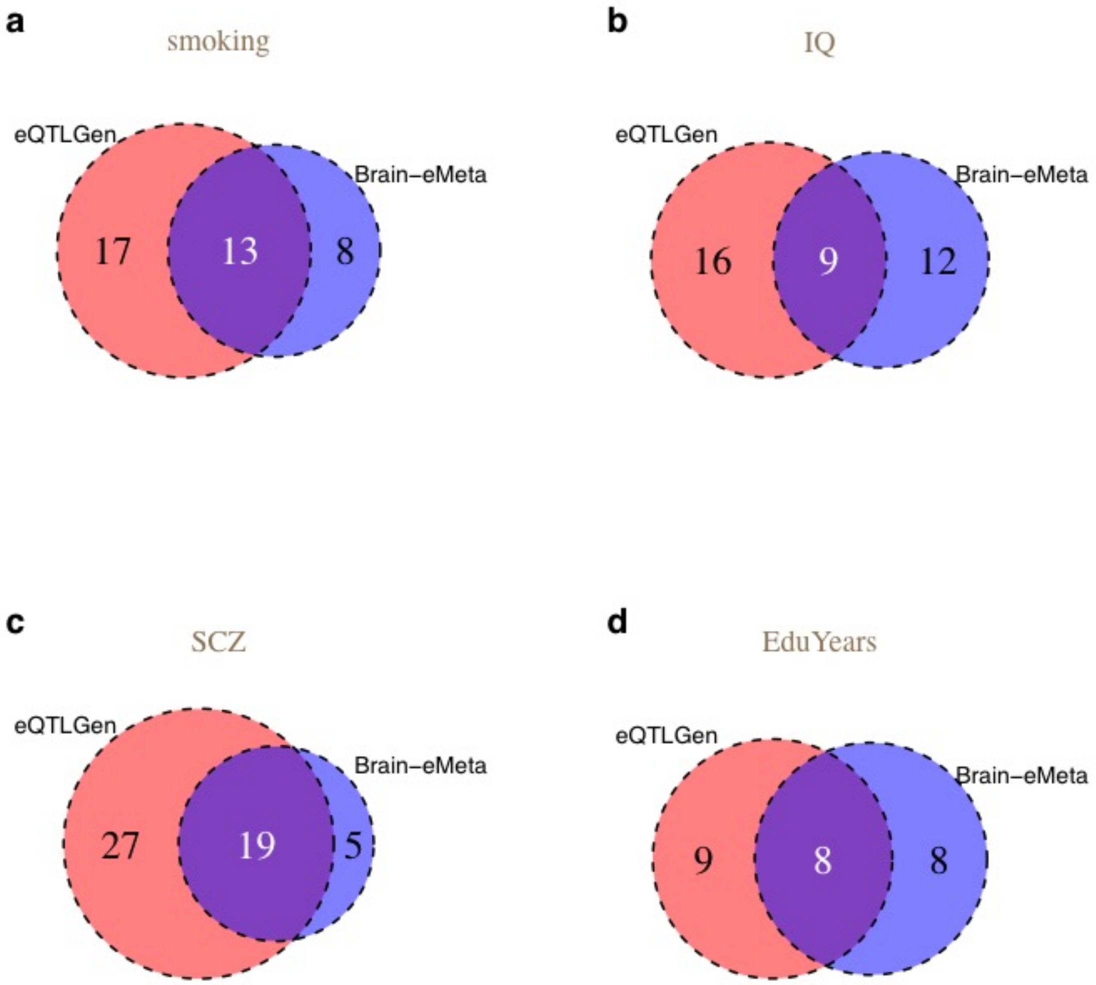

Supplementary Figure 23 Number of genes associated with smoking (**a**), IQ (**b**), SCZ (**c**), and EduYears (**d**) in eQTLGen (blood) and Brain-eMeta (brain). The genes were identified by the SMR analysis using GWAS and eQTL summary data.

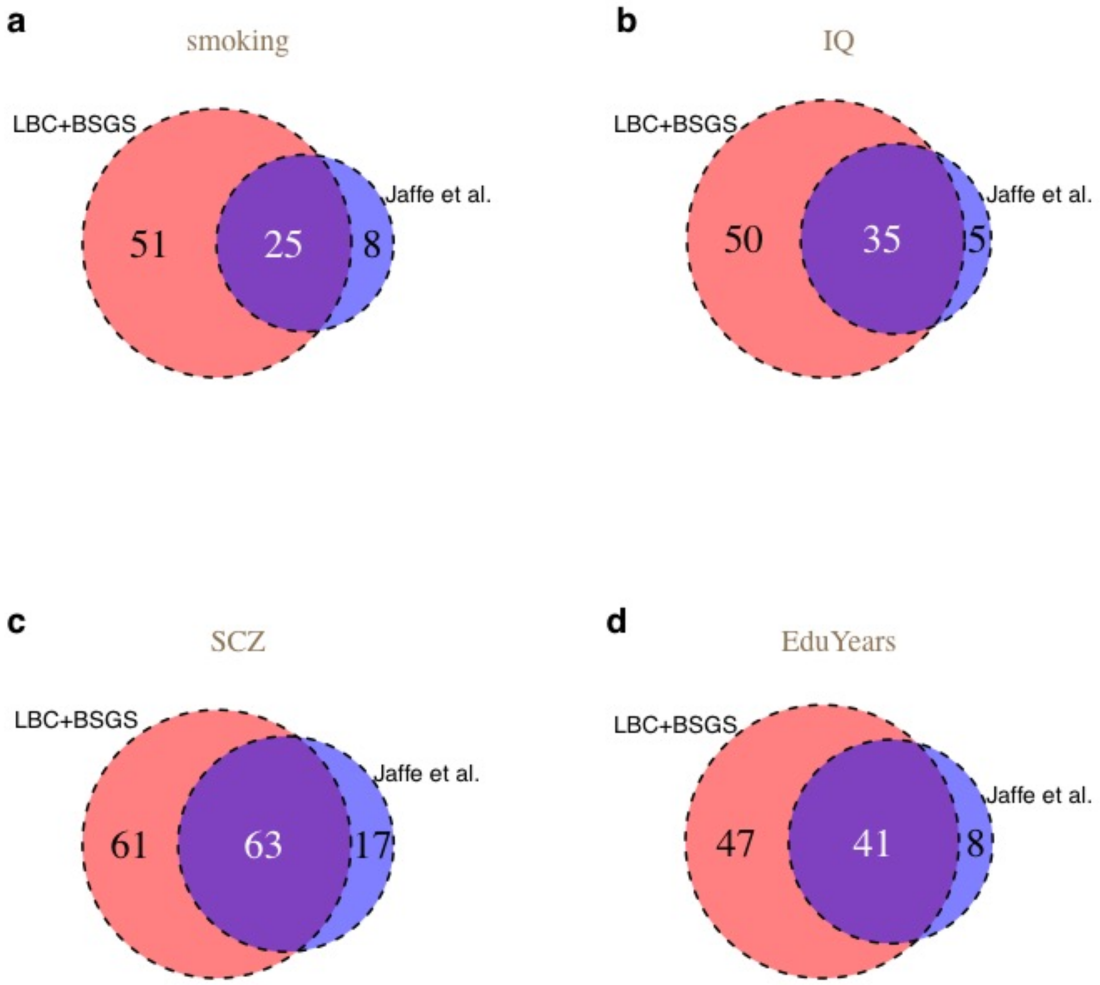

Supplementary Figure 24 Number of DNAm sites associated with smoking (**a**), IQ (**b**), SCZ (**c**), and EduYears (**d**) in LBC+BSGS (blood) and Jaffe et al. (brain). The DNAm sites were identified by the SMR analysis using GWAS and mQTL summary data.

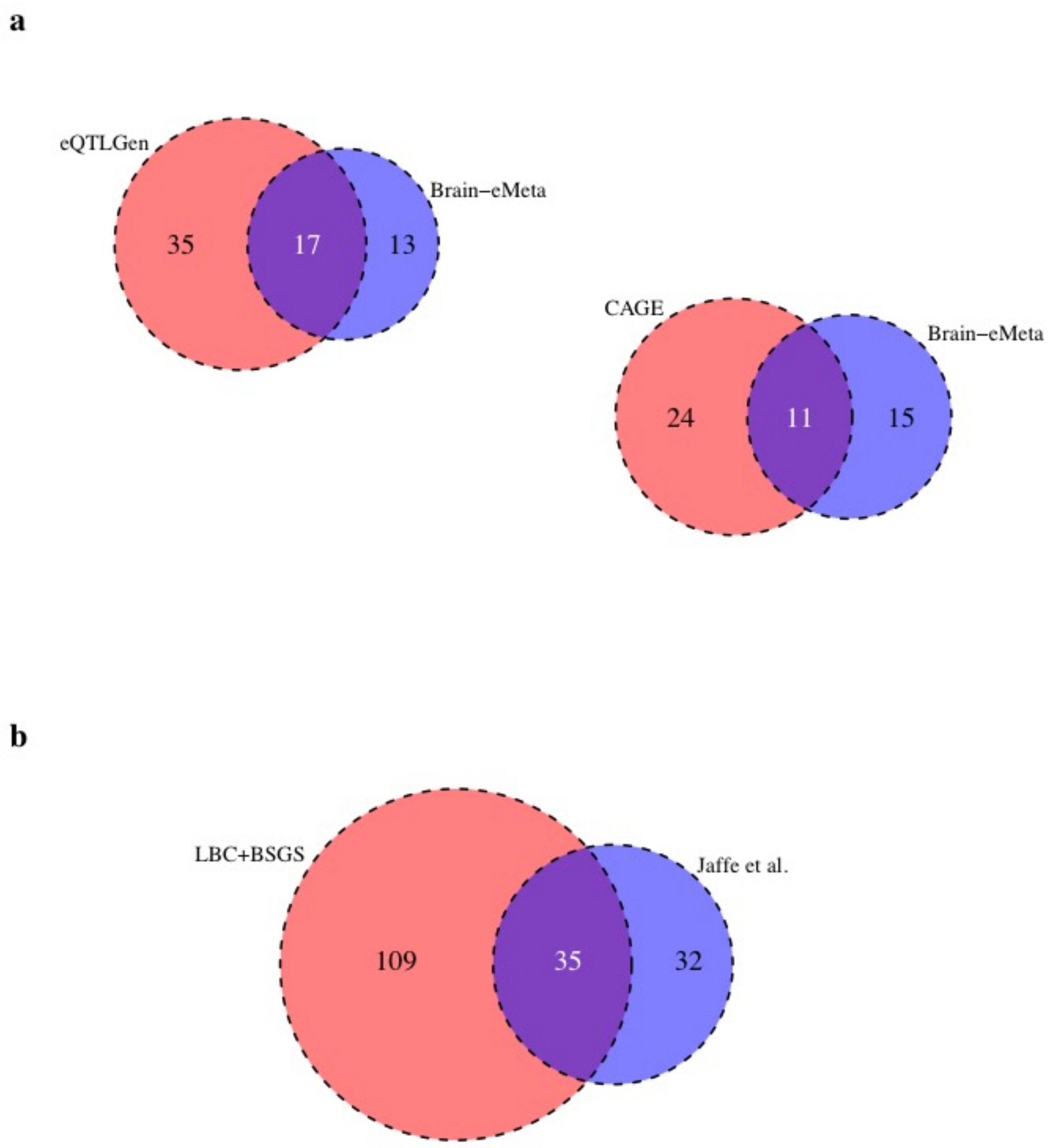

Supplementary Figure 25 Number of genes (**a**) and DNAm sites (**b**) showed pleiotropy effects ($P_{SMR} < 1.8\times10^{-6}$ and $P_{HEIDI} > 0.05$) with 4 brain-related traits by an integrative analysis of GWAS data with eQTL (mQTL) data from brain and blood samples using the SMR & HEIDI approach. The four brain-related traits are smoking, IQ, SCZ and EduYears.

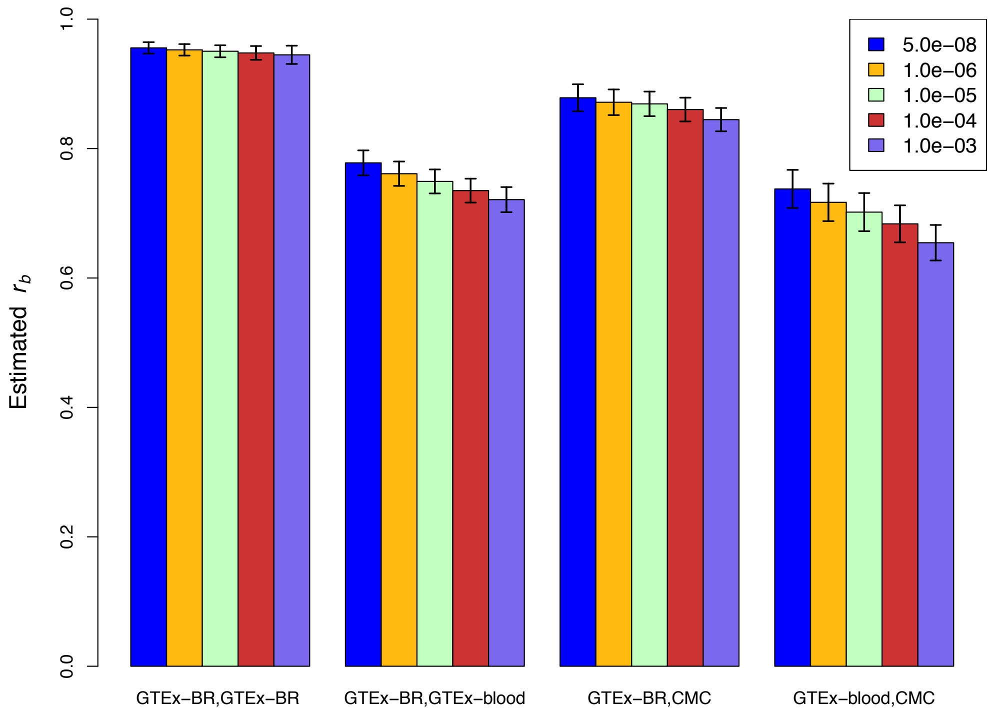

Supplementary Figure 26 Estimates of r_b between two tissues for cis-eQTLs selected at different thresholds from the reference tissue. Each analysis involves three tissues, one tissue as the reference for selecting the top associated cis-eQTLs and the other two tissues for the estimation of r_b. GTEx-muscle was used as the reference tissue to select the top associated cis-eQTLs at 5 different thresholds (i.e. 5.0e-08, 1.0e-06, 1.0e-05, 1.0e-04, and 1.0e-03). GTEx-BR, GTEx-BR: mean estimate of r_b from pairwise brain regions in GTEx. GTEx-BR, GTEx-blood: mean estimate of r_b between blood and 10 brain regions in GTEx. GTEx-BR, CMC: mean estimate of r_b between CMC and 10 brain regions in GTEx. GTEx-blood, CMC: estimate of r_b between GTEx-blood and CMC.

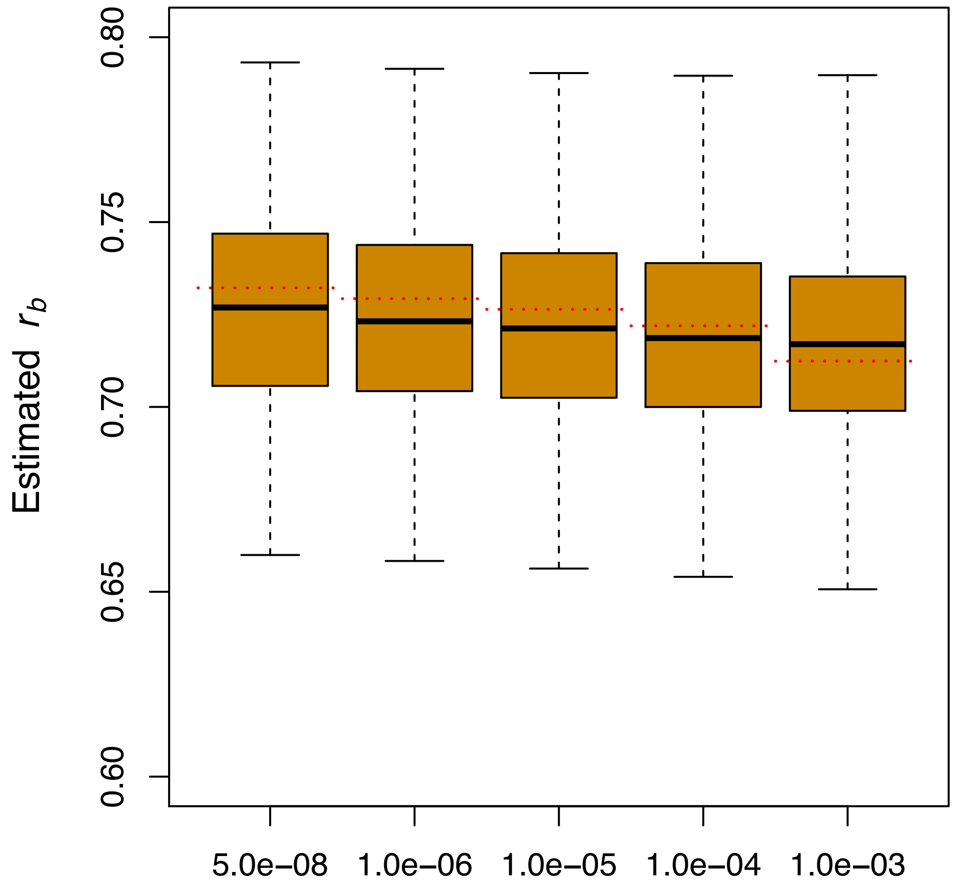

Supplementary Figure 27 Estimates of r_b between two tissues for cis-eQTLs selected at different p-value thresholds in the reference tissue. The gene expression levels in three tissues were simulated based on the UK10K data set with the SNPs in common with HapMap3 (See **Supplementary Note 1** for details). In each simulation replicate, we generated 1,000 probes. The true SNP effects were generated from a multivariate normal distribution with a correlation parameter of 0.7 and the residues in gene expression levels were also simulated from a multivariate normal distribution with a correlation parameter of 0.20 between tissues (**Supplementary Note 1**). The first tissue was used as the reference for selecting the top associated cis-eQTLs at a p-value threshold and the other two tissues were used to estimate r_b at the selected cis-eQTLs. Each box represents the distribution of estimates from 100 simulation replicates. The red dash lines represent the correlation of the true effects generated from the simulation for the corresponding selected probes.

Supplementary Table 1 eQTL summary data

Data set	Tissue	*n*	Data type	*m*	No. of probes and/or genes
GTEx	Brain, anterior cingulate cortex BA24	72	RNA-Seq	5,815,921	23,509
GTEx	Brain, hippocampus	81	RNA-Seq	6,110,317	23,880
GTEx	Brain, hypothalamus	81	RNA-Seq	6,097,172	24,654
GTEx	Brain, putamen basal ganglia	82	RNA-Seq	6,143,910	23,362
GTEx	Brain, cerebellar hemisphere	89	RNA-Seq	6,241,253	24,065
GTEx	Brain, frontal cortex BA9	92	RNA-Seq	6,381,609	24,120
GTEx	Brain, nucleus accumbens basal ganglia	93	RNA-Seq	6,406,794	24,542
GTEx	Brain, cortex	96	RNA-Seq	6,540,080	24,366
GTEx	Brain, caudate basal ganglia	100	RNA-Seq	6,573,031	24,621
GTEx	Brain, cerebellum	103	RNA-Seq	6,554,532	24,762
GTEx	Whole blood	338	RNA-Seq	9,206,530	23,164
CMC[a]	Dorsolateral prefrontal cortex	467	RNA-Seq	1,102,001	14,366
ROSMAP	Brain, cortex	494	RNA-Seq	6,440,707	12,979
Braineac	10 CNS tissues	134	Microarray	6,187,834	25,490
CAGE	Peripheral blood	2,765	Microarray	7,763,174	38,624
eQTLGen	Peripheral blood	14,115	Microarray	10,209,777	44,556

We analyzed eQTL summary data spanning brain and blood from 6 datasets. 10 CNS tissues in Braineac are frontal cortex (FCTX), hippocampus (HIPP), medulla (specifically inferior olivary nucleus, MEDU), occipital cortex (specifically primary visual cortex, OCTX), putamen (PUTM), substantia nigra (SNIG), thalamus (THAL), temporal cortex(TCTX), intralobular white matter (WHMT), and cerebellar cortex (CRBL). For each tissue, we listed the sample size, data type, number of SNPs, and number of probes and/or genes. [a]CMC, only SNP-gene pairs at FDR < 0. 20 were available in the public domain. For the other data sets, we had the full eQTL associations in the cis-regions. ***n***: sample size; ***m***: number of SNPs.

Supplementary Table 2 Number of matched genes out of 4,257 selected from GTEx-muscle between different data sets

Data set 1	Data set 2	No. of matched genes	Data set 1	Data set 2	No. of matched genes
GTEx-brain1	GTEx-brain2	3,726	GTEx-brain5	GTEx-brain6	3,827
GTEx-brain1	GTEx-brain3	3,652	GTEx-brain5	GTEx-brain7	3,782
GTEx-brain1	GTEx-brain4	3,682	GTEx-brain5	GTEx-brain8	3,794
GTEx-brain1	GTEx-brain5	3,735	GTEx-brain5	GTEx-brain9	3,819
GTEx-brain1	GTEx-brain6	3,735	GTEx-brain5	GTEx-brain10	3,771
GTEx-brain1	GTEx-brain7	3,717	GTEx-brain5	GTEx-blood	3,575
GTEx-brain1	GTEx-brain8	3,716	GTEx-brain5	CMC	1,436
GTEx-brain1	GTEx-brain9	3,720	GTEx-brain5	ROSMAP	2,227
GTEx-brain1	GTEx-brain10	3,700	GTEx-brain5	Braineac	2,191
GTEx-brain1	GTEx-blood	3,468	GTEx-brain6	GTEx-brain7	3,775
GTEx-brain1	CMC	1,415	GTEx-brain6	GTEx-brain8	3,788
GTEx-brain1	ROSMAP	2,186	GTEx-brain6	GTEx-brain9	3,804
GTEx-brain1	Braineac	2,142	GTEx-brain6	GTEx-brain10	3,765
GTEx-brain2	GTEx-brain3	3,738	GTEx-brain6	GTEx-blood	3,546
GTEx-brain2	GTEx-brain4	3,776	GTEx-brain6	CMC	1,434
GTEx-brain2	GTEx-brain5	3,827	GTEx-brain6	ROSMAP	2,213
GTEx-brain2	GTEx-brain6	3,809	GTEx-brain6	Braineac	2,176
GTEx-brain2	GTEx-brain7	3,787	GTEx-brain7	GTEx-brain8	3,772
GTEx-brain2	GTEx-brain8	3,809	GTEx-brain7	GTEx-brain9	3,776
GTEx-brain2	GTEx-brain9	3,841	GTEx-brain7	GTEx-brain10	3,751
GTEx-brain2	GTEx-brain10	3,793	GTEx-brain7	GTEx-blood	3,532
GTEx-brain2	GTEx-blood	3,581	GTEx-brain7	CMC	1,430
GTEx-brain2	CMC	1,438	GTEx-brain7	ROSMAP	2,208
GTEx-brain2	ROSMAP	2,227	GTEx-brain7	Braineac	2,174
GTEx-brain2	Braineac	2,192	GTEx-brain8	GTEx-brain9	3,799
GTEx-brain3	GTEx-brain4	3,776	GTEx-brain8	GTEx-brain10	3,763
GTEx-brain3	GTEx-brain5	3,729	GTEx-brain8	GTEx-blood	3,550
GTEx-brain3	GTEx-brain6	3,721	GTEx-brain8	CMC	1,430
GTEx-brain3	GTEx-brain7	3,702	GTEx-brain8	ROSMAP	2,205
GTEx-brain3	GTEx-brain8	3,717	GTEx-brain8	Braineac	2,191
GTEx-brain3	GTEx-brain9	3,732	GTEx-brain9	GTEx-brain10	3,782
GTEx-brain3	GTEx-brain10	3,692	GTEx-brain9	GTEx-blood	3,562
GTEx-brain3	GTEx-blood	3,521	GTEx-brain9	CMC	1,435
GTEx-brain3	CMC	1,425	GTEx-brain9	ROSMAP	2,224
GTEx-brain3	ROSMAP	2,209	GTEx-brain9	Braineac	2,186
GTEx-brain3	Braineac	2,156	GTEx-brain10	GTEx-blood	3,522
GTEx-brain4	GTEx-brain5	3,781	GTEx-brain10	CMC	1,425
GTEx-brain4	GTEx-brain6	3,750	GTEx-brain10	ROSMAP	2,213
GTEx-brain4	GTEx-brain7	3,731	GTEx-brain10	Braineac	2,174
GTEx-brain4	GTEx-brain8	3,744	GTEx-blood	CMC	1,388
GTEx-brain4	GTEx-brain9	3,773	GTEx-blood	ROSMAP	2,209
GTEx-brain4	GTEx-brain10	3,721	GTEx-blood	Braineac	2,526
GTEx-brain4	GTEx-blood	3,569	CMC	ROSMAP	1,043
GTEx-brain4	CMC	1,431	CMC	Braineac	1,113
GTEx-brain4	ROSMAP	2,225	ROSMAP	Braineac	1,354
GTEx-brain4	Braineac	2,177			

We selected the top associated cis-eQTLs at $P_{\mathrm{eQTL}} < 5\times10^{-8}$ for 4,257 genes in GTEx-muscle and matched those selected cis-eQTLs and genes with other data sets. GTEx-brain1 – GTEx-brain10 represent 10 brain regions in GTEx: brain-anterior cingulate cortex BA24, brain-caudate basal ganglia, brain-cerebellar hemisphere, brain-cerebellum, brain-cortex, brain-frontal cortex BA9, brain-hippocampus, brain-hypothalamus, brain-nucleus accumbens basal ganglia, and brain-putamen basal ganglia.

Supplementary Table 3 mQTL summary data

**Data set**	**Tissue**	***n***	***m***	**No. of probes**
ROSMAP[a]	Brain cortical	468	5,211,394	417,700
Hannon et al.[b]	Fetal brain	166	312,180	26,840
Jaffe et al.[c]	Frontal cortex	526	1,544,693	138,917
LBC	peripheral blood	1,366	9,183,310	448,554
BSGS	peripheral blood	614	7,856,389	417,059
LBC+BSGS	peripheral blood	1,980	7,664,968	397,621

All 5 datasets were based on the Illumina HumanMethylation450K array. [a]ROSMAP, only SNPs within 5Kb of the DNAm probes were available; [b]Hannon et al., only SNPs with $P_{\mathrm{mQTL}} < 1\times10^{-10}$ were available; [c]Jaffe et al., only SNPs with FDR < 0.1 (corresponding to $P_{\mathrm{mQTL}} < 8.6\times10^{-4}$) were available; ***n***: sample size; ***m*** : number of SNPs.

Supplementary Table 4 Number of matched DNAm probes between different data sets

Data set 1	Data set 2	No. of matched probes
BSGS	LBC	6,561
BSGS	Jaffe et al.	5,267
BSGS	ROSMAP	5,809
LBC	Jaffe et al.	5,416
LBC	ROSMAP	6,057
Jaffe et al.	ROSMAP	4,892

We selected the top associated cis-mQTLs at $P_{mQTL} < 1\times10^{-10}$ for 26,840 DNAm probes in the data from Hannon et al. and matched those selected cis-mQTLs and DNAm probes with other DNAm data sets.

Supplementary Table 5 *P* value of fold enrichment for tissue-specific mQTLs in each functional category

**Category**	**No. of mQTLs**	**Fold enrichment**	**SE**	***t***	***P* value**
TssA	140	0.630	0.122	-3.033	1.45×10^{-3}
Prom	655	0.916	0.059	-1.424	7.76×10^{-2}
Tx	546	1.035	0.081	0.432	3.33×10^{-1}
TxWk	331	1.155	0.124	1.25	1.06×10^{-1}
TxEn	254	1.570	0.181	3.149	9.17×10^{-4}*
EnhA	99	1.675	0.258	2.616	5.15×10^{-3}
EnhW	250	1.416	0.168	2.476	6.97×10^{-3}
DNase	75	1.663	0.405	1.637	5.29×10^{-2}
ZNFRpts	15	0.876	0.296	-0.419	3.41×10^{-1}
Het	57	0.835	0.162	-1.018	1.56×10^{-1}
PromP	40	0.682	0.211	-1.507	6.99×10^{-2}
PromBiv	168	0.869	0.151	-0.867	1.94×10^{-1}
ReprPC	353	0.757	0.074	-3.284	5.65×10^{-4}
Quies	2436	0.924	0.029	-2.621	4.42×10^{-3}

t = (fold enrichment - 1)/SE; P value is estimated form t-distribution. The red asterisk indicated significant enrichment of T_D after the correction for multiple testing ($P < 0.05/14$).

Supplementary Table 6 Summary data of GWAS

Phenotype	n	n_{case}	$n_{control}$	No. of SNPs
SCZ	150,064	36,989	113,075	9,444,231
EduYears	293,723	/	/	8,146,841
smoking	453,693	208,988	244,705	7,288,503
IQ	146,819	/	/	7,288,503

We included 4 brain-related complex traits in the analysis. GWAS summary statistics for SCZ and EduYears were from the latest meta-analyses, and summary data for smoking and IQ were from GWAS analysis in the latest release of the UK Biobank (**Methods**). n: sample size; n_{case}: number of cases; $n_{control}$: number of controls.

Supplementary Table 7 Replication rate of top eQTLs selected from muscle in different tissues or datasets

Data set	Tissue	n	m	5×10^{-8}	π_1
GTEx	Brain, Anterior cingulate cortex BA24	72	3,740	0.115	0.578
GTEx	Brain, hippocampus	81	3,810	0.107	0.599
GTEx	Brain, hypothalamus	81	3,860	0.116	0.599
GTEx	Brain, putamen basal ganglia	82	3,801	0.131	0.625
GTEx	Brain, cerebellar hemisphere	89	3,759	0.178	0.650
GTEx	Brain, frontal cortex BA9	92	3,844	0.148	0.622
GTEx	Brain, nucleus accumbens basal ganglia	93	3,871	0.148	0.626
GTEx	Brain, cortex	96	3,831	0.171	0.657
GTEx	Brain, caudate basal ganglia	100	3,884	0.162	0.649
GTEx	Brain, cerebellum	103	3,852	0.210	0.700
GTEx	Whole blood	338	3,821	0.292	0.715
CMC	Dorsolateral Prefrontal Cortex	467	2,024	0.528	0.988
Braineac	aveALL[a]	134	2,275	0.056	0.424

[a]aveALL represents eQTLs associated with average gene expression across 10 brain regions in Braineac. $\boldsymbol{n}$**:** sample size; $\boldsymbol{m}$**:** number of cis-eQTLs in common with those selected from GTEx-muscle; $\mathbf{5\times10^{-8}}$**:** replication rate at $P < 5\times10^{-8}$; $\boldsymbol{\pi_1}$ (the proportion of true positive) was estimated using the method described in Storey et al.[7].

Supplementary Note 1: Simulation studies

We performed a series of simulations based on a whole-genome sequencing data from the UK10K project[1]. Details of the data and quantify control can be found elsewhere[1]. For simplicity, we limited the analysis to SNPs on chromosome 22 and those in common with HapMap3[8], and further excluded SNPs with MAF < 0.01 or Hardy-Weinberg Equilibrium (HWE) P value < 1×10^{-6}. There were 16,805 SNPs and 3,642 unrelated individuals included in the simulation studies.

To investigate the unbiasedness of r_b method

We performed simulations to investigate the unbiasedness of the r_b method. To this end, we randomly sampled a position on chromosome 22 and defined a ± 2Mb window centered on the position as a cis-region. We randomly sampled a SNP in the cis-region as the causal variant. The genetic effects of the causal variant in three tissues (one tissue was used for selecting top associated cis-eQTLs, and the other two were used for estimating r_b) were drawn from a multivariate normal distribution, $\mathbf{b}{\sim}MVN(\mathbf{0},\begin{bmatrix}1 & \rho_{12} & \rho_{13}\\ \rho_{12} & 1 & \rho_{23}\\ \rho_{13} & \rho_{23} & 1\end{bmatrix})$, with ρ being the correlation of SNP effects between tissues. Correlation of estimation error (r_e) may occur due to sample overlap and phenotype correlation, and therefore we generated residual error ($\mathbf{e}$) in three tissues from a multivariate normal distribution, $\mathbf{e}{\sim}MVN(\mathbf{0},\mathbf{S})$, where $\mathbf{S}$ is the variance-covariance matrix. The ij-th element of $\mathbf{S}$ is $S_{ij} = r_e\sqrt{\text{var}(e_i)\text{var}(e_j)}$, where $\text{var}(e_i) = 2p(1-p)b_i^2(\frac{1}{q_i^2}-1)$ with p being the MAF, b_i being the effect size of the causal variant in tissue i, and q_i^2 being the proportion of variance in expression level of a gene explained by the causal variant. Five levels of r_e (0.1, 0.3, 0.5, 0.7, 0.9) were considered. Thus, gene expression levels in the three tissues for each of 3,642 individuals in our sample can be generated from a linear model $\mathbf{Y} = \mathbf{Xb} + \mathbf{e}$. eQTL effect size and SE in cis-region were estimated by a linear regression analysis of the simulated gene expression level for each SNP in each tissue. We repeated this process for 2,000 times to mimic the data for 2,000 genes. We then repeated the whole simulation with 100 replications for each level of r_e.

To investigate the unbiasedness of the MeCS method

To test performance of MeCS, we also conducted extensive simulations based on UK10K data under the null and alternative hypotheses pertaining to eQTL effect. We randomly sampled a gene position and a causal variant in cis-region using the same method as above (**Supplementary Note 1**). We set $b = 1, q^2 = 0.01$, and simulated $b_i = b + d_i$, where b is the mean SNP effect across all tissues, and d_i is the deviation of SNP effect from b in tissue i, $d_i{\sim}N(0,0.1)$. For simplicity, we assumed that there are only 2 tissues. We can generate the expression level of a gene in the 2 tissues by a simple additive model $\mathbf{Y} = \mathbf{Xb} + \mathbf{e}$ with different levels of θ, where $\mathbf{e}$ is

generated from a multivariate normal distribution, $\mathbf{e} \sim MVN\left(\mathbf{0}, \begin{bmatrix} \mathrm{var}(e_i) & \theta\sqrt{\mathrm{var}(e_i)\mathrm{var}(e_j)} \\ \theta\sqrt{\mathrm{var}(e_i)\mathrm{var}(e_j)} & \mathrm{var}(e_j) \end{bmatrix}\right)$. We then performed simple regression analyses to estimate eQTL effect sizes and SE for each SNP in each tissue. Furthermore, a null model (i.e. $b = d_i = 0$) was used to assess type I error. Each simulation was replicated 1,000 times.

Supplementary Note 2: Estimating effective sample size

We know from Yang et al.[9] that the effective sample size (n_{eff}) can be calculated as

$$n_{\mathrm{eff}} = (\chi^2 - 1)\frac{1 - q^2}{q^2}$$

where q^2 is the proportion of variance in gene expression explained by the cis-eQTL. We selected the top cis-eQTLs from GTEx-blood at $P < 5\times10^{-8}$, and calculated the mean χ^2 value of these SNPs across 10 brain regions in GTEx. Assuming that q^2 is similar across all brain regions, n_{eff} of the meta-analyzed GTEx-brain data can be estimated from the following equation

$$\frac{n_{\mathrm{eff}_{\mathrm{GTEx-brain}}}}{\bar{n}_{\mathrm{brain\_region}}} = \frac{(\chi^2 - 1)_{\mathrm{GTEx-brain}}}{\overline{(\chi^2 - 1)}_{\mathrm{brain\_region}}}$$

where $\bar{n}_{\mathrm{Brain}}$ is the mean sample size across all brain regions, and $\overline{(\chi^2 - 1)}_{\mathrm{brain\_region}}$ is the mean of mean χ^2 values across all brain regions.

Supplementary Note 3: Acknowledgements

HRS (dbGaP accession: phs000428.v1.p1): HRS is supported by the National Institute on Aging (NIA U01AG009740). The genotyping was funded separately by the National Institute on Aging (RC2 AG036495, RC4 AG039029). Genotyping was conducted by the NIH Center for Inherited Disease Research (CIDR) at Johns Hopkins University. Genotyping quality control and final preparation of the data were performed by the Genetics Coordinating Center at the University of Washington.

CMC (Synapse accession: syn2759792): CMC data were generated as part of the CommonMind Consortium supported by funding from Takeda Pharmaceuticals Company Limited, F. Hoffman-La Roche Ltd and NIH grants R01MH085542, R01MH093725, P50MH066392, P50MH080405, R01MH097276, RO1-MH-075916, P50M096891, P50MH084053S1, R37MH057881 and R37MH057881S1, HHSN271201300031C, AG02219, AG05138 and MH06692. Brain tissue for the study was obtained from the following brain bank collections: the Mount Sinai NIH Brain and Tissue Repository, the University of Pennsylvania Alzheimer's Disease Core Center, the

University of Pittsburgh NeuroBioBank and Brain and Tissue Repositories and the NIMH Human Brain Collection Core. CMC Leadership: Pamela Sklar, Joseph Buxbaum (Icahn School of Medicine at Mount Sinai), Bernie Devlin, David Lewis (University of Pittsburgh), Raquel Gur, Chang-Gyu Hahn (University of Pennsylvania), Keisuke Hirai, Hiroyoshi Toyoshiba (Takeda Pharmaceuticals Company Limited), Enrico Domenici, Laurent Essioux (F. Hoffman-La Roche Ltd), Lara Mangravite, Mette Peters (Sage Bionetworks), Thomas Lehner, Barbara Lipska (NIMH).

GTEx (dbGaP accession: phs000424.v6.p1): The Genotype-Tissue Expression (GTEx) Project was supported by the Common Fund of the Office of the Director of the National Institutes of Health (commonfund.nih.gov/GTEx). Additional funds were provided by the NCI, NHGRI, NHLBI, NIDA, NIMH, and NINDS. Donors were enrolled at Biospecimen Source Sites funded by NCI Leidos Biomedical Research, Inc. subcontracts to the National Disease Research Interchange (10XS170), Roswell Park Cancer Institute (10XS171), and Science Care, Inc. (X10S172). The Laboratory, Data Analysis, and Coordinating Center (LDACC) was funded through a contract (HHSN268201000029C) to the The Broad Institute, Inc. Biorepository operations were funded through a Leidos Biomedical Research, Inc. subcontract to Van Andel Research Institute (10ST1035). Additional data repository and project management were provided by Leidos Biomedical Research, Inc.(HHSN261200800001E). The Brain Bank was supported supplements to University of Miami grant DA006227. Statistical Methods development grants were made to the University of Geneva (MH090941 & MH101814), the University of Chicago (MH090951, MH090937, MH101825, & MH101820), the University of North Carolina - Chapel Hill (MH090936), North Carolina State University (MH101819), Harvard University (MH090948), Stanford University (MH101782), Washington University (MH101810), and to the University of Pennsylvania (MH101822)

UK10K (EGA accessions: EGAS00001000108 and EGAS00001000090): The UK10K project was funded by the Wellcome Trust award WT091310. Twins UK (TUK): TUK was funded by the Wellcome Trust and ENGAGE project grant agreement HEALTH-F4- 2007–201413. The study also receives support from the Department of Health via the National Institute for Health Research (NIHR)-funded BioResource, Clinical Research Facility and Biomedical Research Centre based at Guy's and St. Thomas' NHS Foundation Trust in partnership with King's College London. Dr Spector is an NIHR senior Investigator and ERC Senior Researcher. Funding for the project was also provided by the British Heart Foundation grant PG/12/38/29615 (Dr Jamshidi). A full list of the investigators who contributed to the UK10K sequencing is available from http://www.UK10K.org.

UKB: This study has been conducted using UK Biobank resource under Application Number 12514. UK Biobank was established by the Wellcome Trust medical charity, Medical Research Council, Department of Health, Scottish Government and the Northwest Regional Development Agency. It has also had funding from the Welsh Assembly Government, British Heart Foundation and Diabetes UK.

Supplementary References

1. UK10K Consortium. The UK10K project identifies rare variants in health and disease. *Nature* **526**, 82-90 (2015).
2. Lin, Y. *et al.* Housekeeping genes, revisited at the single-cell level. *bioRxiv*, 229815 (2017).
3. Eisenberg, E. & Levanon, E.Y. Human housekeeping genes, revisited. *Trends in Genetics* **29**, 569-574 (2013).
4. Yang, J. *et al.* Conditional and joint multiple-SNP analysis of GWAS summary statistics identifies additional variants influencing complex traits. *Nature genetics* **44**, 369-375 (2012).
5. Yang, J., Lee, S.H., Goddard, M.E. & Visscher, P.M. GCTA: a tool for genome-wide complex trait analysis. *The American Journal of Human Genetics* **88**, 76-82 (2011).
6. Fromer, M. *et al.* Gene expression elucidates functional impact of polygenic risk for schizophrenia. *Nature neuroscience* **19**, 1442-1453 (2016).
7. Storey, J.D. & Tibshirani, R. Statistical significance for genomewide studies. *Proceedings of the National Academy of Sciences* **100**, 9440-9445 (2003).
8. International HapMap 3 Consortium. Integrating common and rare genetic variation in diverse human populations. *Nature* **467**, 52-58 (2010).
9. Yang, J. *et al.* Genomic inflation factors under polygenic inheritance. *European Journal of Human Genetics* **19**, 807-812 (2011).
